# MUC15 Ectodomain Architecture Regulates Integrin Clustering to Control Cancer Metastasis

**DOI:** 10.1002/advs.202513552

**Published:** 2025-10-13

**Authors:** Simei Zhang, Hongyuan Zhu, Zeen Zhu, Shuai Wu, Yiqun Song, Jin Wang, Xinlong Chen, Weikun Qian, Jianpeng Li, Yangyang Yue, Qinhong Xu, Zhiping Ruan, Qing Li, Yaomin Zhu, Tian Jian Lu, Guy M. Genin, Feng Xu, Zheng Wang, Min Lin

**Affiliations:** ^1^ Department of Hepatobiliary Surgery The First Affiliated Hospital of Xi'an Jiaotong University Xi'an 710061 P. R. China; ^2^ Pancreatic Disease Treatment Center of Xi'an Jiaotong University Xi'an 710061 P. R. China; ^3^ Department of Anesthesiology & Center for Brain Science The First Affiliated Hospital of Xi'an Jiaotong University Xi'an 710061 P. R. China; ^4^ The Key Laboratory of Biomedical Information Engineering of Ministry of Education, School of Life Science and Technology Xi'an Jiaotong University Xi'an 710049 P. R. China; ^5^ Bioinspired Engineering and Biomechanics Center (BEBC) Xi'an Jiaotong University Xi'an 710049 P. R. China; ^6^ Department of Cardiovascular Surgery The First Affiliated Hospital of Xi'an Jiaotong University Xi'an 710061 P. R. China; ^7^ Department of Vascular Surgery The First Affiliated Hospital of Xi'an Jiaotong University Xi'an 710061 P. R. China; ^8^ Department of Geriatric Surgery The First Affiliated Hospital of Xi'an Jiaotong University Xi'an 710061 P. R. China; ^9^ Department of Medical Oncology The First Affiliated Hospital of Xi'an Jiaotong University Xi'an 710061 P. R. China; ^10^ State Key Laboratory of Mechanics and Control of Mechanical Structures Nanjing University of Aeronautics and Astronautics Nanjing 210016 P. R. China; ^11^ Department of Mechanical Engineering & Materials Science Washington University in St. Louis St. Louis MO 63130 USA; ^12^ NSF Science and Technology Center for Engineering Mechanobiology Washington University in St. Louis St. Louis MO 63130 USA; ^13^ Department of General Surgery Huashan Hospital Fudan University 12 Urumqi Road (M) Shanghai China

**Keywords:** cancer metastasis, ECM remodeling, glycocalyx, integrins, mechanotransduction

## Abstract

Cancer metastasis is governed by physical cues at the cell‐matrix interface, with matrix stiffness, ligand density, and topography established as key determinants. Here, a fourth critical factor in cancer metastasis, the architecture of the cell‐surface glycocalyx is identified. Using MUC15 as a representative small glycoprotein, mathematical modeling and domain truncation experiments are combined to show that glycoprotein size distribution governs integrin adhesion states and metastatic outcomes. MUC15 localizes to focal adhesions and interact with integrins, while larger glycoproteins such as MUC1 are sterically excluded. These physical effects, rather than intracellular signaling, dictate adhesion state transitions: removing MUC15's ectodomain eliminated its anti‐metastatic effects, whereas removal of its cytoplasmic tail has no effect. In in vivo pancreatic cancer models, modulating MUC15 levels controlled metastasis as predicted by the mathematical model. These findings establish glycocalyx architecture as a mechanical regulator of cancer progression and suggest therapeutic strategies targeting glycoprotein size distribution.

## Graphical abstract

This study uncovers a size‐dependent adhesion paradigm, demonstrating that the glycocalyx protein MUC15 suppresses pancreatic cancer progression by attenuating integrin activation, focal adhesion assembly, and YAP mechanotransduction. Integrating computational modeling and experimental validation, the work reveals a mechanical mechanism by which MUC15 constrains metastatic behavior, offering new insights into glycocalyx‐mediated cancer regulation.

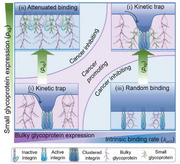

## 1 Introduction

The cell‐surface glycocalyx, a dense polymer coating of glycoproteins on animal cells, creates a steric barrier between transmembrane receptors and the extracellular matrix (ECM). Its physical dimensions regulate integrin‐mediated adhesion and mechanotransduction, for instance, studies of glycoprotein ectodomains have shown that longer glycoproteins attenuate receptor‐ligand binding.^[^ ^1^, ^2^, ^3^ ^]^ In particular, when the glycocalyx is dominated by large glycoproteins, steric hindrance reduces the baseline binding affinity between cell‐surface integrins and ECM proteins, allowing integrins to diffuse more freely in the membrane.^[^ ^4^ ^]^

However, once an integrin binds to its ECM ligand, it locally compresses the glycocalyx and pulls the adjacent membrane closer to the ECM. This compression creates a zone of enhanced receptor‐ligand binding affinity, effectively funneling diffusing integrins into the site and driving their clustering,^[^ ^1^, ^5^, ^6^, ^7^, ^8^ ^]^ a phenomenon known as the “kinetic trap”. This effect drives focal adhesion maturation ^[^ ^2^, ^3^ ^]^ and is implicated in the metastatic cascade.^[^ ^9^ ^]^ While this framework effectively explains the behavior of larger glycoproteins (>1000 amino acids),^[^ ^10^, ^11^, ^12^ ^]^ it cannot fully account for why a shorter glycoprotein like MUC15 (334 amino acids) has opposite effects on cell migration in different contexts. In particular, MUC15 promotes development of gastric,^[^ ^13^ ^]^ colon,^[^ ^14^ ^]^ brain,^[^ ^15^ ^]^ and thyroid cancer,^[^ ^16^ ^]^ but inhibits hepatocellular carcinoma ^[^ ^17^ ^]^ and prostate,^[^ ^18^ ^]^ cervical,^[^ ^19^ ^]^ renal,^[^ ^20^ ^]^ and liver cancer.^[^ ^21^ ^]^ These paradoxical outcomes are not readily reconciled by a purely physical framework.

To address this paradox, we conducted a systematic investigation of glycoprotein size effects through integrated experimental and computational approaches. Building on well‐established methods for probing mechanical roles of mucins,^[^ ^1^, ^2^, ^3^, ^22^ ^]^ we employed domain truncation experiments to directly manipulate the physical dimensions of MUC15 while preserving its other functional domains. This approach enabled us to isolate size‐specific effects from other potential mechanisms and directly test whether the MUC15 ectodomain's physical dimensions, rather than just its biochemical properties, determine its functional effects.

We then tested these ideas in both cell culture and mouse models of pancreatic ductal adenocarcinoma (PDAC). Through mathematical modeling and experimental validation, we identified three distinct integrin adhesion states governed by glycoprotein size distribution, expression levels, and integrin binding kinetics. Using both tail vein injection and syngeneic orthotopic transplantation models, we demonstrated that modulating MUC15 levels effectively regulated tumor metastasis and ECM remodeling, highlighting its potential as a therapeutic target for metastatic control. These findings thus not only establish glycoprotein size and architecture as key physical regulators of tumor progression but also highlight glycoprotein size distribution as a potential therapeutic handle for metastatic control.

## 2 Results

### 2.1 MUC15 Downregulation Correlates with PDAC Malignancy and Enhanced Tumor Progression

**Figure 1:**
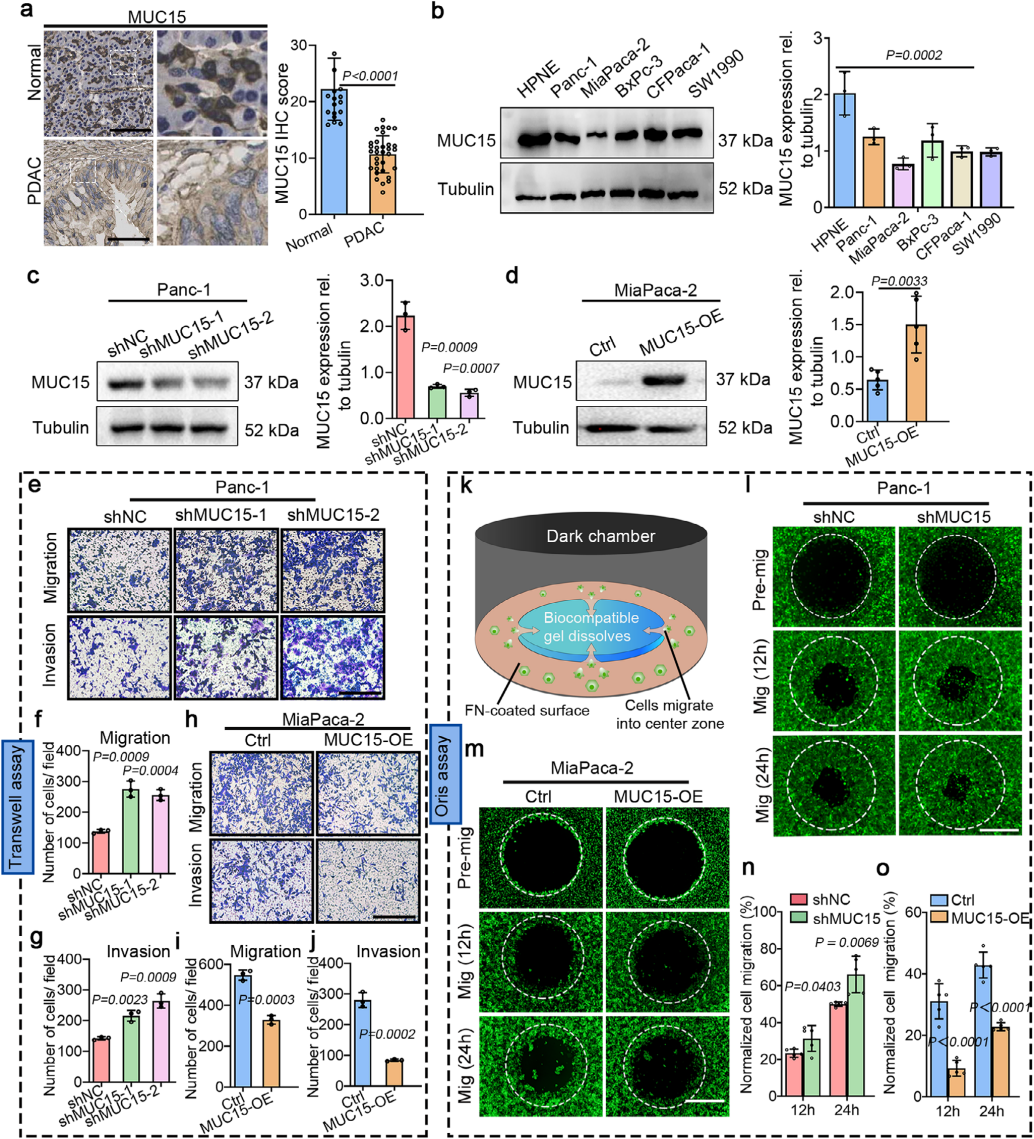
MUC15 downregulation correlates with PDAC malignancy. a) MUC15 immunohistochemistry (IHC) in normal pancreatic tissues (*n* = 20) and PDAC tissues (*n* = 70) (left) with quantification of MUC15 IHC score (right). Statistical analysis by paired two‐tailed Student's *t*‐test. b) Western blotting analysis of MUC15 expression levels in pancreatic duct epithelium cell line (HPNE) and various PDAC cell lines (Panc‐1, MiaPaca‐2, BxPc‐3, CFPaca‐1, SW1990) (*n =* 3) (left) and quantification of MUC15 expression levels relative to HPNE (right). Data from three independent experiments, analyzed by one‐way ANOVA. c) Left: Western blotting analysis of MUC15 knockdown efficiency in Panc‐1 cell line. Right: Quantification of MUC15 expression levels relative to shNC. d) Western blotting analysis of MUC15 overexpression efficiency in MiaPaca‐2 cell line (left) and quantification of MUC15 expression level relative to control (right). e) Images of Matrigel transwell migration and invasion assays in MUC15‐depleted Panc‐1 cells. f,g) Quantification of the number of cells per field in (e). h) Images of Matrigel transwell migration and invasion assays in MUC15‐overexpressed MiaPaca‐2 cells. i,j) Quantification of the number of cells per field in (h). k) Schematic diagram of the Oris migration assay, where cells migrate inward to fill a circular gap created by a physical barrier. l,m) Representative time‐course images of Oris migration assays in MUC15‐depleted Panc‐1 cells (l) and in MUC15‐overexpressed MiaPaCa‐2 cells (m). Dashed lines indicate the boundary of the initial cell‐free zone at the start of the assay (0 h). n,o) Quantification of wound‐healing area relative to the wound area at 0 h. Data were collected from three independent experiments (e, h, l, m). Data are presented as mean ± s.e.m., and *P* values were obtained using unpaired two‐tailed Student's *t*‐test (a, c, d, f, g, i, j, n, o) and one‐way ANOVA followed by Tukey's post hoc test (b). Scale bars: 100 µm (a, e, h, l, m).

To assess whether MUC15 expression correlates with malignancy in PDAC, we analyzed MUC15 expression levels in both PDAC and normal human pancreatic tissues. MUC15 expression analysis in pancreatic tissues revealed a characteristic pattern: normal pancreatic tissue showed robust MUC15 expression, while PDAC tumors showed significantly reduced expression (**Figure** **1**a). This pattern was confirmed at the mRNA level through public dataset analysis (Figure S1a–c, Supporting Information). Similarly, PDAC cell lines (Panc‐1, MiaPaca‐2, BxPc‐3, CFPaca‐1, and SW1990) showed markedly reduced MUC15 expression compared to normal pancreatic ductal (HPNE) cells (Figure 1b; Figure S1d, Supporting Information).

To evaluate the correlation between MUC15 expression and migration/invasion in PDAC cells, we employed a complementary experimental approach using multiple cell lines with different baseline MUC15 expression levels. We downregulated MUC15 in naturally high‐expressing cell lines (Panc‐1 and BxPc‐3), where a substantial reduction could be achieved (Figure 1c; Figure S2, Supporting Information). Conversely, overexpressing MUC15 in a low‐expressing cell line (MiaPaca‐2), where physiologically relevant increases could be obtained (Figure 1d; Figure S3, Supporting Information). This strategy allowed us to 1) observe MUC15's effects across a broad range of expression levels, 2) avoid potential artifacts from extreme overexpression or incomplete knockdown, and 3) demonstrate the generalizability of MUC15's roles across different PDAC cellular contexts through complementary manipulation of protein levels in multiple cell lines. Through the strategy, we found that MUC15 depletion led to significantly elongated cells with a greater number of thin membrane protrusions (Figure S2d, Supporting Information). In comparison, MUC15 overexpression resulted in the opposite trends (Figure S3b, Supporting Information).

To comprehensively assess the effects of MUC15 on cell motility and invasiveness, we employed three complementary migration assays: transwell, Oris migration, and wound healing assays (Figure 1e–o; Figure S4, Supporting Information). Each assay provided distinct insights into cell behavior. In transwell assays, which measure both migration and invasion of cells, MUC15 downregulation in Panc‐1 and BxPc‐3 cells significantly increased the number of cells traversing the membrane (Figure 1e–g; Figure S4a, Supporting Information). Conversely, MUC15 overexpression in MiaPaca‐2 cells markedly reduced transmembrane migration (Figure 1h–j). The Oris migration assay, which tracks cells moving into a defined circular gap (Figure 1k), revealed that MUC15‐depleted cells migrated more rapidly toward the center (Figure 1l,n), while MUC15‐overexpressing cells showed significantly reduced migration rates (Figure 1m,o). Wound healing assays further confirmed these findings: MUC15 knockdown accelerated wound closure (Figure S4b,c, Supporting Information), while MUC15 overexpression delayed it (Figure S4d, Supporting Information). Taken together, these three independent assays demonstrated that MUC15 consistently regulates PDAC cell migration and invasion. MUC15 depletion promoted these processes (Figure 1e–g,l,n; Figure S4a–c, Supporting Information), while MUC15 overexpression inhibited them (Figure 1h–j,m,o; Figure S4d, Supporting Information).

To evaluate whether MUC15 affects PDAC cell proliferation, we performed 5‐ethynyl‐2′‐deoxyuridine (EdU) incorporation assays, which measure DNA synthesis during cell division by detecting nucleoside analog incorporation into newly synthesized DNA. After optimizing labeling conditions, we compared EdU incorporation rates between control and MUC15‐modified cells. Neither MUC15 downregulation in Panc‐1 and BxPc‐3 cells nor MUC15 overexpression in MiaPaca‐2 cells significantly altered the percentage of EdU‐positive cells (Figure S5, Supporting Information). This lack of effect on DNA synthesis rates, combined with our migration findings, suggests that MUC15's primary function in PDAC cells is regulating cell motility and invasion rather than proliferation. These results help distinguish MUC15's specific role in cancer progression, suggesting that it primarily influences metastatic potential through effects on cell migration rather than tumor growth through changes in proliferation.

For the following detailed mechanistic studies, we selected the Panc‐1‐shMUC15‐2 cell line for MUC15 knockdown experiments based on superior knockdown efficiency at both mRNA and protein levels (Figure 1c; Figure S2a,b, Supporting Information), along with more consistent single‐cell migration behavior compared to BxPc‐3 cells, which tend to form clusters that complicate migration analysis (Figure 1e; Figure S4a, Supporting Information). While both cell lines showed robust MUC15 knockdown, the reduced cell‐cell clustering in Panc‐1 cells allowed us to more precisely isolate MUC15's effects on individual cell migration and invasion.

### 2.2 MUC15 Inhibits YAP Nuclear Localization Independently of Hippo Pathway

**Figure 2:**
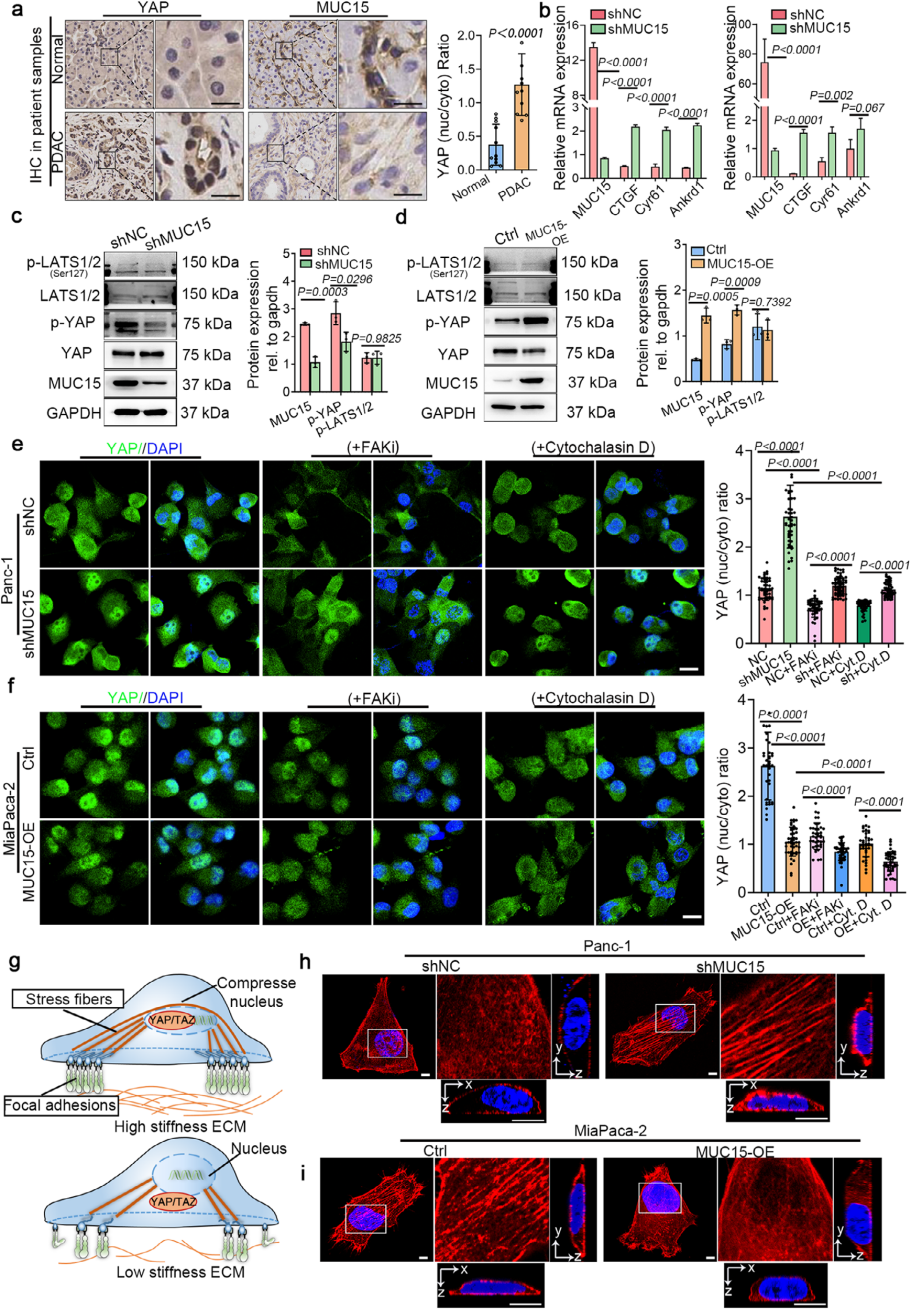
MUC15 inhibits YAP nuclear translocation through direct mechanotransduction that is independent of the Hippo pathway. a) Immunohistochemistry staining of MUC15 and YAP in normal and PDAC tissues. b) Expression of YAP‐target genes (CTGF, Cyr61, and Ankrd1) in Panc‐1 cells (h) and BxPc‐3 cells (i) with and without MUC15 depletion, analyzed by qRT‐PCR using 18S as endogenous control (*n* = 3). c,d) Immunoblotting analysis of pS127‐LATS1/2 and p‐YAP (left) with corresponding quantification (right). e) Immunostaining of YAP after treatment with FAK or F‐actin inhibitors in Panc‐1 cells with or without MUC15 depletion (left) and quantification of YAP nuclear‐to‐cytoplasmic ratios (right). Data from three independent experiments; *n* = 48 (shNC), 45 (shMUC15), 46 (shNC + FAKi), 58 (shMUC15 + FAKi), 46 (shNC + cytochalasin D), and 56 (shMUC15 + cytochalasin D) cells. f) Immunostaining of YAP after treatment with FAK or F‐actin inhibitors in MiaPaca‐2 cells with or without MUC15 overexpression (left) and quantification of YAP nuc/cyto ratios (right). Data from three independent experiments; *n* = 37 (Ctrl), 46 (MUC15‐OE), 39 (Ctrl + FAKi), 44 (MUC15‐OE + FAKi), 38 (Ctrl + cytochalasin D), and 44 (MUC15‐OE + cytochalasin D) cells. g) Schematic of F‐actin tension‐induced nuclear deformation and YAP localization. h,i) F‐actin organization and nuclear morphology in Panc‐1 cells with or without MUC15 depletion (h) and in MiaPaca‐2 cells with or without MUC15 overexpression (i). Insets show detailed F‐actin organization. Cross‐sectional views taken along XZ‐ and YZ‐planes through the nuclear center. Statistical analyses: unpaired two‐tailed Student's *t*‐test (a, b, c, d) and one‐way ANOVA (e, f). Scale bars: 100 µm (a), 50 µm (e, f), 2 µm (h, i).

The promotion of migration and invasion by MUC15 depletion contradicts the widespread reports of attenuation of cancer cell migration and invasion by glycocalyx downregulation.^[^ ^22^, ^23^ ^]^ To identify the underlying pathway, we first investigated YAP, a downstream effector of the Hippo pathway whose activation promotes cell adhesion, migration, invasion, and tumor progression.^[^ ^24^ ^]^ A potential role of YAP was supported by immunohistochemical staining of pancreatic cancer tissues, which showed increased nuclear localization of YAP in comparison to normal pancreatic tissues (**Figure** **2**a). Furthermore, a negative correlation was observed between YAP nuclear localization and MUC15 expression levels (Figure 2a). A potential regulatory effect was further supported by the observation of upregulated YAP‐target genes, including connective tissue growth factor (CTGF),^[^ ^25^ ^]^ cysteine‐rich angiogenic inducer (Cyr61),^[^ ^26^ ^]^ ankyrin repeat domain 1 (Ankrd1),^[^ ^27^ ^]^ in both MUC15‐depleted Panc‐1 and BxPc‐3 cells (Figure 2b).

We hypothesized that this increase in YAP activity was regulated by the Hippo pathway, and tested this hypothesis by measuring how YAP signaling in these cell lines correlated with activity of the Hippo signaling kinases LATS1/2. LATS1/2 regulates YAP activity by phosphorylating serine residues,^[^ ^28^ ^]^ and we therefore expected that the alterations in the activity of LATS1/2 kinases correlated with changes in YAP activity in cell lines with MUC15‐depleted or overexpression. While Western blot assays revealed significant changes in YAP phosphorylation in response to MUC15 levels, they showed negligible changes in the activity of LATS1/2 kinases (Figure 2c,d). These findings contradicted our initial hypothesis and suggest that MUC15 regulates YAP activity through a Hippo pathway‐independent mechanism.

### 2.3 MUC15 Inhibits YAP Signaling Through Attenuation of Mechanotransduction

We hypothesized that the inhibition of YAP signaling by MUC15 was instead associated with integrin dynamics and the effects of these dynamics on cytoskeletal mechanotransduction, as elucidated in our prior studies.^[^ ^29^, ^30^ ^]^ This hypothesis was motivated by the observation that the glycocalyx regulates integrin dynamics by acting as a physical barrier between cells and ECM.^[^ ^4^, ^22^ ^]^ To test this hypothesis, we quantified YAP localization in MUC15‐deleted Panc‐1 cells and found that MUC15 depletion increased YAP nucleus localization. Treatment with a focal adhesion kinase inhibitor (FAKi) or an F‐actin inhibitor (cytochalasin D) partially reversed this effect (Figure 2e). In contrast, MUC15 overexpression in MiaPaCa‐2 cells reduced YAP nuclear localization, with a greater reduction observed following treatment with either FAKi or cytochalasin D (Figure 2f). These results supported our hypothesis, indicating the involvement of focal adhesion assembly and actin stress fibers in the regulation of YAP activity by MUC15.

Motivated by observations from the literature that perinuclear stress fiber contractility induces nuclear deformation,^[^ ^29^, ^30^, ^31^ ^]^ thereby facilitating nuclear localization of YAP (Figure 2g), we thus explored whether MUC15 affects how perinuclear stress fibers deform the nucleus. Depleting MUC15 in Panc‐1cells increased perinuclear stress fiber organization and nuclear deformation (Figure 2h; Figure S6a, Supporting Information). In contrast, overexpressing MUC15 in MiaPaca‐2 cells led to disrupted perinuclear stress fibers and decreased nuclear deformation (Figure 2i; Figure S6b, Supporting Information). These results suggest that MUC15 depletion activates YAP signaling through mechanotransduction via the adhesion‐cytoskeleton‐nucleus machinery.

### 2.4 MUC15 Inhibits Focal Adhesion Assembly and Signaling

**Figure 3:**
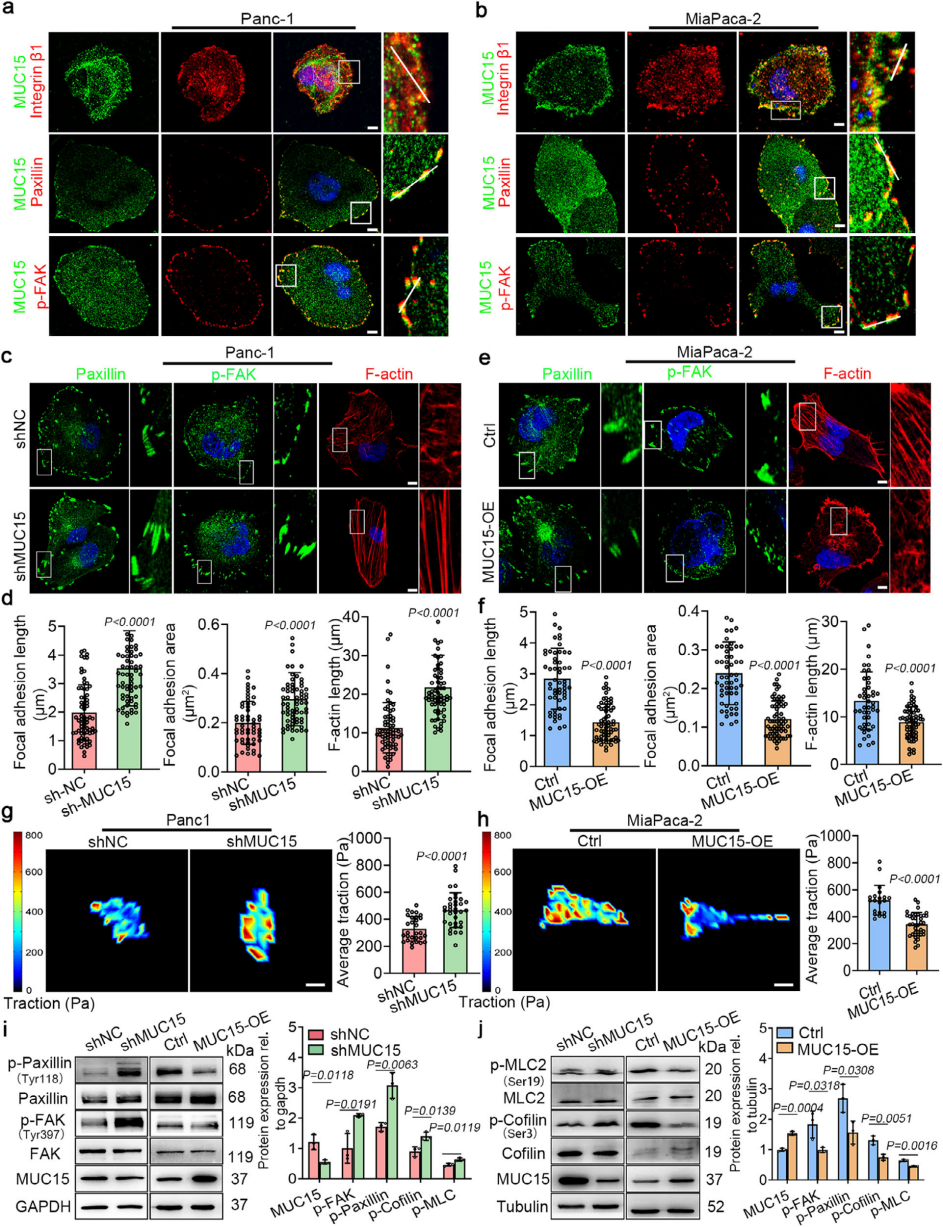
Effects of MUC15 on focal adhesions assembly and signaling. a,b) Immunostaining of MUC15, integrin β1, paxillin, and p‐FAK in Panc‐1 and MiaPaca‐2 cells. c,d) Immunostaining of paxillin, p‐FAK, F‐actin, and DAPI in shRNA/shNC‐transfected Panc‐1 cells and corresponding quantification of the length and the area of focal adhesions as well as the F‐actin length. Each data point represents an independent cell or focal adhesion; *n* = 69 (shNC) and 67 (shMUC15) cells for focal adhesion length; *n* = 55 (shNC) and 67 (shMUC15) cells for focal adhesion area; *n* = 68 (shNC) and 67 (shMUC15) cells for F‐actin length. e,f) Immunostaining of paxillin, p‐FAK, F‐actin, and DAPI in MUC15 lentivirus and control‐transfected MiaPaca‐2 cells and corresponding quantification of the length and the area of focal adhesions as well as the F‐actin length. *n* = 56 (Ctrl) and 67 (MUC15‐OE) cells for focal adhesion length; 55 (Ctrl) and 67 (MUC15‐OE) cells for focal adhesion area; *n* = 45 (Ctrl)) and *n* = 64 (MUC15‐OE) cells for F‐actin length. g,h) Representative heat maps of traction stress in shRNA/shNC‐transfected Panc‐1 cells and MUC15 lentivirus and control‐transfected MiaPaca‐2 cells, respectively. The corresponding quantification of average traction stress per cell is shown on the right; *n* = 29 (shNC) and 34 (shMUC15) for Panc‐1 cells; *n* = 20 (Ctrl) and 33 (MUC15‐OE) for MiaPaca‐2 cells. i,j) Western blot analyses of p‐Paxillin, p‐FAK, p‐MLC2, and p‐Cofilin in Panc‐1 and MiaPaca‐2 cells after shRNA and MUC15 lentivirus transfection. The corresponding quantification of p‐FAK, p‐Paxillin, p‐MLC2, and p‐Cofilin relative to shNC and control cells, respectively, is shown on the right. Each data point represents an independent experiment, and the experiment was repeated three times. *P* values were obtained using an unpaired two‐tailed Student's *t*‐test (d‐j). Scale bars: 20 µm (a‐e), 30 µm (g‐h).

To further characterize how MUC15 affects YAP signaling via the adhesion‐cytoskeleton‐nucleus axis, we examined interactions between MUC15 and key components of focal adhesions. Immunostaining showed that MUC15 had a baseline diffuse presence across the cell membrane, while showing enrichment at the cell periphery and at focal adhesions, as shown by co‐localization with integrin β1, paxillin, and p‐FAK (**Figure** **3**a,b; Figure S7, Supporting Information). This distribution pattern was consistent across both the Panc‐1 and MiaPaCa‐2 cell lines, regardless of whether MUC15 levels were modified. Co‐immunoprecipitation experiments confirmed direct interaction between MUC15 and integrin β1 (Figure S8, Supporting Information). In contrast, the larger glycoprotein MUC1 is excluded from focal adhesions due to a kinetic trap mechanism,^[^ ^2^ ^]^ wherein bulky glycoproteins are physically prevented from entering the narrow adhesive cleft. The short ectodomain of MUC15 is sufficiently small to enter and be retained within this confined zone, enabling it to co‐localize with integrins. The ability of MUC15 to remain within the kinetic trap, while maintaining lateral membrane mobility, suggests a size‐dependent regulatory mechanism by which glycoproteins differentially influence focal adhesion assembly and downstream signaling during cancer progression.

While MUC15 depletion in Panc‐1 cells increased the length and size of focal adhesions (Figure 3c,d). MUC15 overexpression in MiaPaCa‐2 cells reduced these (Figure 3e,f). These results demonstrated that MUC15 localizes to focal adhesions and inhibits focal adhesion growth in these pancreatic cancer cells. Larger focal adhesions, as observed in MUC15‐depleted Panc‐1 cells, are often associated with enhanced assembly of the actin cytoskeleton and with increased cellular contractility,^[^ ^32^, ^33^ ^]^ both of which promote cancer cell migration and invasion through increased ECM remodeling.^[^ ^34^ ^]^ Consistent with this, immunostaining of F‐actin showed that MUC15 depletion led to increased assembly of F‐actin (Figure 3c,d), while MUC15 overexpression had the opposite effect (Figure 3e,f). Traction force microscopy performed on cells seeded on top of a polyethylene glycol hydrogel modified with RGD peptide (Figure S9, Supporting Information) showed that downregulating MUC15 in Panc‐1 cells increased cellular tractions (Figure 3g), whereas upregulating MUC15 in MiaPaCa‐2 cells reduced them (Figure 3h). These observations demonstrate that MUC15 downregulates cellular contractility by suppressing the growth of focal adhesion.

To determine the effects of MUC15 on focal adhesion signaling cascades, we characterized the expression of the downstream focal adhesion signaling molecules phosphorylated focal adhesion kinase (p‐FAK), p‐paxillin, phospho‐S19‐myosin light chain 2 (p‐MLC2), and phospho‐S3‐cofilin (p‐cofilin) (Figure 3i,j). The phosphorylation levels of these molecules were upregulated or downregulated by suppressing or overexpressing MUC15, respectively, in accordance with the changes of focal adhesion size (Figure 3c,e). Taken together, these results suggest that MUC15 suppresses focal adhesion assembly and signaling, which reduces cellular contractility, nuclear deformation, and ultimately YAP signaling.

### 2.5 MUC15 Suppresses Integrin β1 Activation and Focal Adhesion Formation

**Figure 4:**
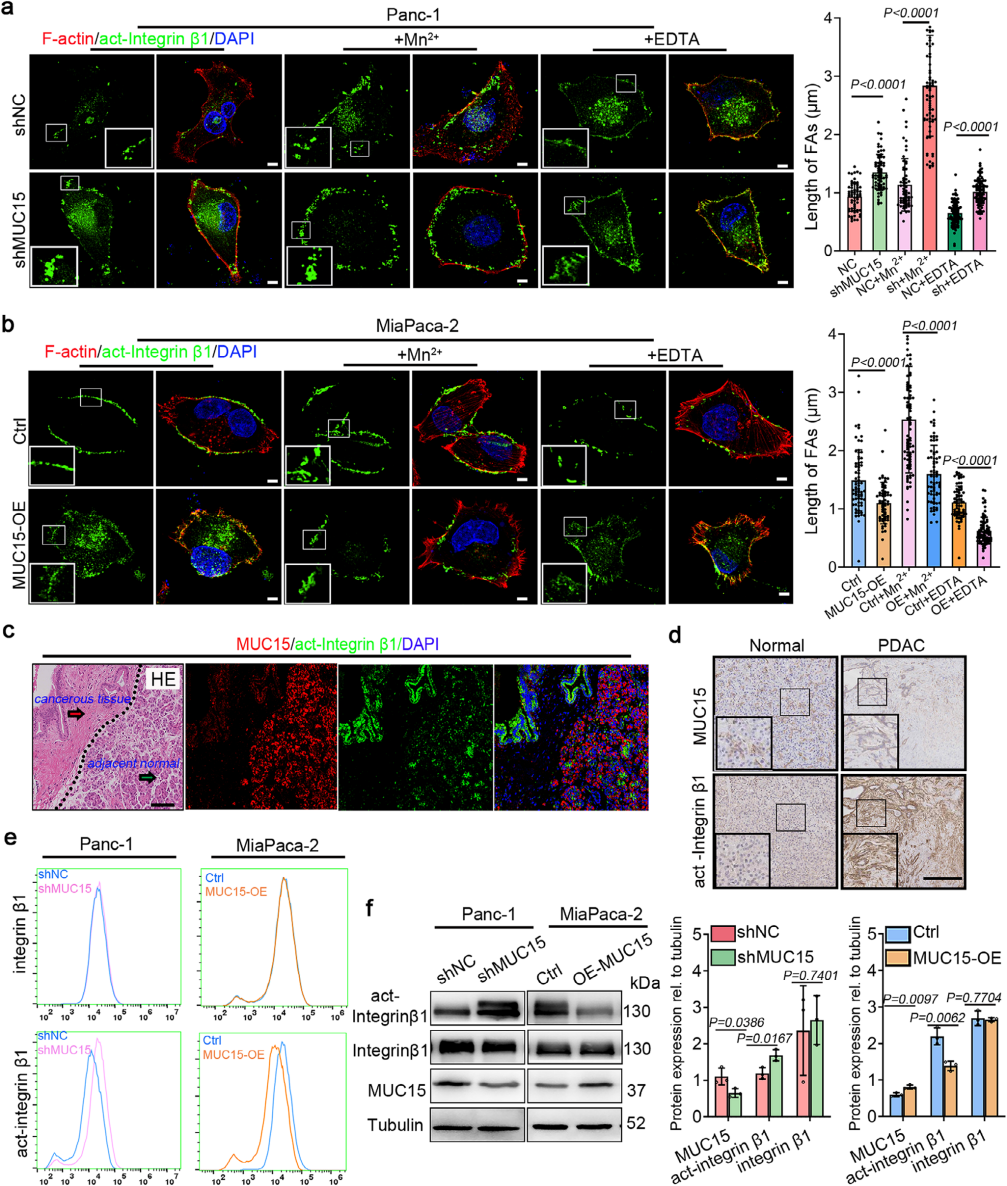
MUC15 suppresses integrin β1 activation and focal adhesion formation. a) Immunostaining of active integrin β1 in Panc‐1 cells expressing control (shNC) or MUC15 shRNA, with or without 30‐min treatment with 1 mM MnCl_2_ or 2 mM EDTA (left). Quantification of focal adhesion lengths (right). Data from three independent experiments; *n* = 64 (shNC), 67 (shMUC15), 63 (shNC + Mn^2^⁺), 75 (shMUC15 + Mn^2^⁺), 92 (shNC + EDTA), and 86 (shMUC15 + EDTA) cells. b) Immunostaining of active integrin β1 in MiaPaca‐2 cells expressing control or MUC15 lentivirus, with or without 30‐min treatment with 1 mM MnCl_2_ or 2 mM EDTA (left). Quantification of focal adhesion lengths (right). Data from three independent experiments; *n* = 73 (Ctrl), 61 (MUC15‐OE), 86 (Ctrl + Mn^2^⁺), 72 (MUC15‐OE + Mn^2^⁺), 70 (Ctrl + EDTA), and 77 (MUC15‐OE + EDTA) cells. c) Haematoxylin and eosin (H&E) staining and immunostaining of MUC15 and active integrin β1 in PDAC tissue. The cancerous tissue area shows the pancreatic intra‐epithelial neoplasia (PIN) on the left (red arrow) of the dashed line, and the para‐cancerous tissue area appears on the right (green arrow). d) Serial tissue sections (normal and PDAC) analyzed by immunohistochemistry staining of MUC15 and activated integrin β1 at the same location. e) Flow cytometry analysis of total integrin β1 and activated integrin β1 on the cell surface of Panc‐1 cells after shRNA/shNC transfection or on MiaPaca‐2 cells with MUC15 lentivirus or control transfection. f) Immunoblotting analysis of total integrin β1 and activated integrin β1 on cells. The corresponding quantification level of total integrin β1 and activated integrin β1 relative to shNC and control cells, respectively. Each data point represents an independent experiment, and the experiment was repeated three times. Each data point represents individual cells from three independent experiments. Statistical analyses were performed using an unpaired two‐tailed Student's *t*‐test (a, b, f). Scale bars: 50 µm (a, b) and 100 µm (c, d).

The above results led us to hypothesize that MUC15 affects focal adhesion growth by interfering with integrin dynamics. To test this hypothesis, we performed immunofluorescence analysis of focal adhesions. MUC15 depletion in Panc‐1 cells significantly increased both the number and size of active integrin β1‐containing adhesions (**Figure** **4**a; Figure S10a, Supporting Information), reaching levels comparable to those induced by Mn^2^⁺ treatment, which forces integrins into their high‐affinity conformation.^[^ ^7^ ^]^ Conversely, MUC15 overexpression reduced focal adhesion formation, as quantified by both metrics (Figure 4b; Figure S10b, Supporting Information).

To confirm whether these observations hold true in vivo, we studied human PDAC tissues using H&E staining to demarcate the cancerous pancreatic intraepithelial neoplasia from the adjacent normal and para‐cancerous tissue (Figure 4c). Immunostaining showed significantly decreased MUC15 expression within the cancerous tissue area, accompanied by increased levels of activated integrin β1 (Figure 4c). Similarly, the analysis of serial tissue sections revealed elevated levels of activated integrin β1 and reduced levels of MUC15 in PDAC tissues compared to normal pancreatic tissues (Figure 4d). These findings provide an in vivo support for the hypothesis that MUC15 inhibits integrin activation and suggest that this inhibition is associated with cancer progression.

To check that our measurements truly showed downregulation of integrin β1 activation, and were not an artifact arising from reduction of the total amounts of integrin β1, we performed flow cytometry analysis of total‐ and activated integrin β1. We observed that neither depletion nor overexpression of MUC15 altered the total level of integrin β1, but that MUC15 depletion increased levels of activated integrin β1, while MUC15 overexpression decreased them (Figure 4e). This observation was further confirmed by immunoblotting analysis, which demonstrated that depletion or overexpression of MUC15 increased or decreased the activation level of integrin β1, respectively, while the total expression level of integrin β1 remained unchanged (Figure 4f). These findings suggest that MUC15 negatively regulates the activation of integrin β1, while keeping the expression level of total integrin β1unchanged.

### 2.6 MUC15 Inhibits Cell Migration and Integrin Clustering Due to Its Size

To explore why the effects of MUC15 on cancer progression can be diametrically opposed to those of larger glycoproteins, we hypothesized that the glycoprotein size distribution determines the effect of the glycocalyx on integrin activation and dynamics. We then tested the hypothesis by developing a mathematical model and validating it against experimental observations. The mathematical model considered a glycocalyx with two populations of glycoproteins of different sizes, both of which interfere with integrin binding.

**Figure 5:**
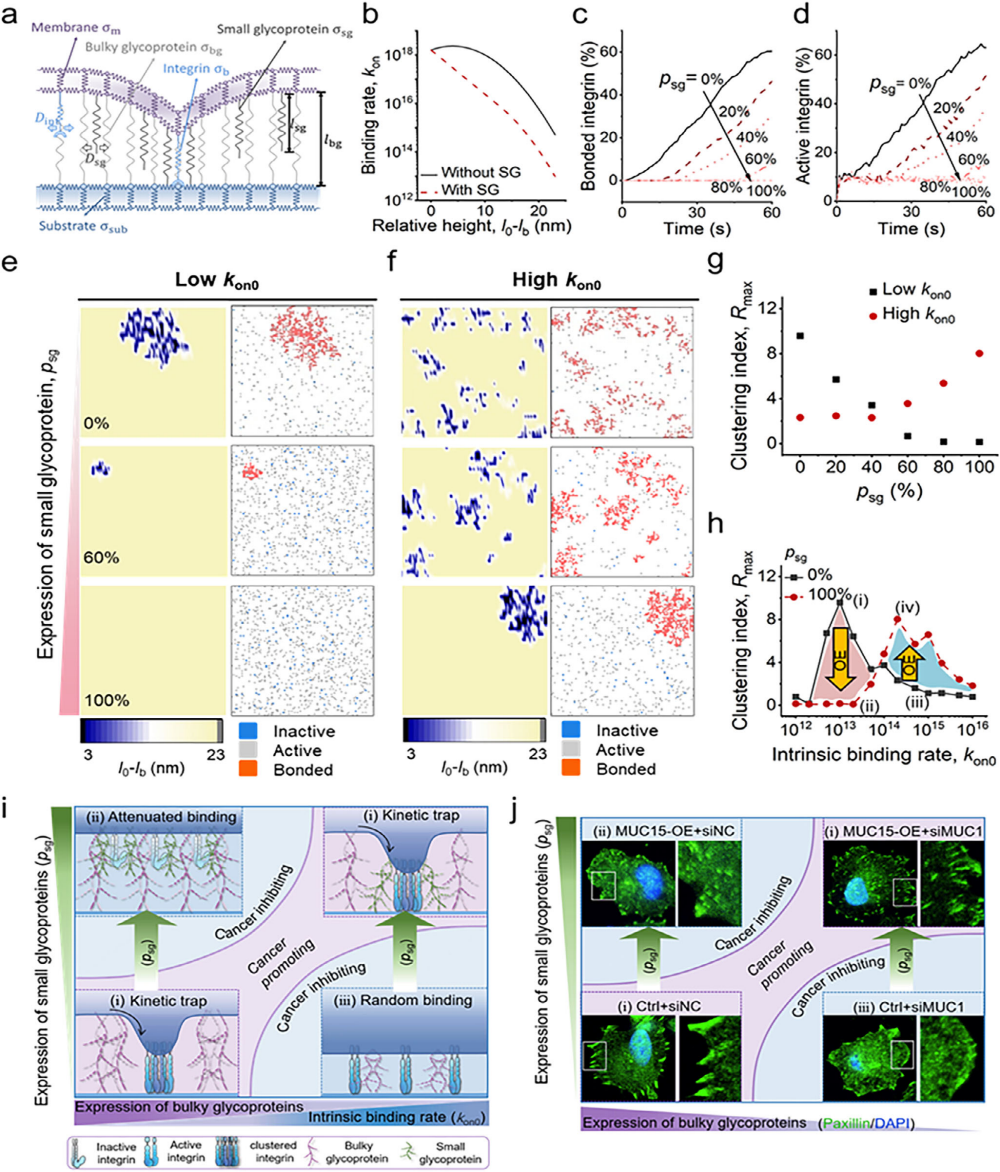
A mathematical model predicting three distinct integrin binding states based upon glycoprotein size and expression. a) A schematic of the model for the mechanical effect of small glycoproteins on integrin‐mediated cell‐ECM adhesions. b) Predicted integrin binding rate as a function of relative membrane height. Black solid line: binding rates in the absence of small glycoprotein; red dashed line: binding rates considering the effect of small glycoprotein. c,d) Predicted kinetic profile plots of integrin bond formation (c) and activation (d) at a given intrinsic binding rate (*k*_on0_ = 10^13^ s^−1^). e,f) Predicted steady‐state distributions of the relative membrane height (left column) and integrin binding states (right column) with increasing expression level of small glycoproteins at (e) a relatively low intrinsic binding rate (*k*_on0_ = 10^13^ s^−1^) and (f) a relatively high intrinsic binding rate (*k*_on0_ = 2 · 10^14^ s^−1^). g,h) Predicted clustering index (*R*_max_) as a function of (g) small glycoprotein expression (*p*_sg_) and (h) intrinsic binding rate with (red) or without (black) small glycoproteins, revealing that overexpression of small glycoproteins can either suppress integrin clustering at low intrinsic binding rate, or promote it at high intrinsic binding rate. i,j) A schematic phase diagram of the predicted effect of small glycoprotein expression (*p*_sg_) and intrinsic binding rate (*k*_on0_) on integrin binding states. Integrin clustering was reduced by attenuated integrin binding (state ii) for high *p*_sg_ and low *k*_on0_, and by random integrin binding (state iii) for low *p_sg_* and high *k*_on0_. The kinetic trap mechanism upregulated integrin clustering at either low *p_sg_* and *k*_on0_ or high *p_sg_* and *k*_on0_. j, Immunofluorescence imaging verified that manipulating glycoprotein expression switched MiaPaca‐2 cells between different binding states.

Building on the framework established by Paszek et al.,^[^ ^2^, ^3^ ^]^ our model represents the system as three coupled elastic networks. The cell membrane and substrate were modeled as independent spring networks, with the glycocalyx layer represented as a third set of interposing springs between them. Within this framework, integrins were simulated as individual spring‐like elements that can diffuse across the cell membrane (**Figure** **5**a; Figure S11, Supporting Information). A key feature of our model was the distinct treatment of different glycoprotein types based on their mechanical properties. Bulky glycoproteins were represented by long, fixed springs, while small glycoproteins like MUC15 were modeled as shorter springs capable of diffusion. This distinction allowed us to predict the mechanical effects of different glycocalyx components on cell‐substrate interactions. The model calculated integrin binding rates by considering the total elastic energy of the system, incorporating contributions from membrane deformation, substrate elasticity, and glycoprotein compression/extension. This mechanical framework thus enabled us to predict how glycoproteins of different sizes influence integrin clustering and subsequent cellular behaviors. The complete mathematical formulation and parameter values are provided in the Supplementary Note.

These small glycoproteins attenuated the intrinsic integrin binding rate (*k*_on0_) of the co‐localized integrin through a steric effect (Figure 5b). We then simulated how varying proportions of small glycoproteins (*p*_sg_) regulate integrin dynamics and relative membrane height (*l*_0_ − *l*_b_) at a fixed intrinsic integrin binding rate (Figure 5c‐g), and then calculated the proportions of bound and active integrins over time (Figure 5c,d). Consistent with our experimental findings that MUC15 suppresses integrin activation and clustering (Figures 3c,e and 4a,b), we observed that increasing *p*_sg_ inhibited integrin activation and clustering at a low intrinsic integrin binding rate, *k*_on0_. This effect was evident from distributions of relative membrane height (lower relative membrane height value indicates higher integrin binding) and integrin activation and clustering (clustering index *R*_max_, see definition in Supplementary Note) (Figure 5e,g).

This inhibitory effect was also consistent with the effect of small glycoproteins (e.g., MUC15) in resisting the development of certain cancers,^[^ ^17^, ^18^, ^20^, ^21^, ^35^ ^]^ yet contrasted sharply with their role in promoting the development of other cancers.^[^ ^13^, ^15^, ^36^ ^]^ To investigate why small glycoproteins act differently in distinct cell types, we tested the hypothesis that small glycoproteins affect integrin clustering differently depending upon the intrinsic integrin binding rate, *k*_on0_. *k*_on0_ varies with cell‐ECM interactions involving actin binding proteins such as talin ^[^ ^37^ ^]^; cell membrane factors such as glycocalyx thickness,^[^ ^2^ ^]^ cadherin density,^[^ ^30^ ^]^ and membrane curvature ^[^ ^23^ ^]^; and properties of the ECM such as stiffness ^[^ ^38^ ^]^ and ligand density.^[^ ^39^ ^]^ Specifically, we proposed that when *k*_on0_ is sufficiently high, small glycoproteins inhibit the kinetic trap mechanism. To test this hypothesis, we increased *k*_on0_, and observed distinct changes in the distribution of relative membrane height and of integrin activation and clustering for a prescribed value of *p*_sg_ (Figure 5f,g). The integrin clustering index showed a biphasic trend, with clustering peaked at low *k*_on0_ and attenuated at high *k*_on0_, which was shifted by the presence of small glycoproteins (Figure 5h). This indicates that overexpression of glycoproteins can either promote or inhibit integrin clustering, thereby creating conditions that may promote or inhibit cancer progression, respectively.

Results revealed three distinct cell adhesion states determined by the interplay between the proportion of the small glycoproteins (*p*_sg_) and the instinct integrin binding rate (*k*_on0_), with the latter being a function of the density of bulky glycoproteins (Figure 5i). State (***i***), with low *k*_on0_ and low *p*_sg_, creates conditions that may promote cancer progression: bound integrins form a kinetic trap that recruits neighboring integrins and promotes integrin clustering. State (***ii***), with low *k*_on0_ and high *p*_sg_, may inhibit cancer progression: overexpression of small glycoproteins interferes with integrin binding to the substrate and thereby inhibits integrin clustering. State (***iii***), with high *k*_on0_ and low *p*_sg_, may also inhibit cancer progression: preferential binding of integrins to the substrate reduces the pool of integrins available to diffuse into clusters. State (***i***) is recovered at high *k*_on0_ and high *p*_sg_, with overexpression of small glycoproteins promoting the kinetic trap mechanism. Thus, overexpression of small glycoprotein can yield diametrically opposite changes on integrin clustering and signaling depending on the integrin binding rate.

To verify the model‐predicted context‐dependent effects of small glycoproteins on integrin clustering, we used MUC1 knockdown to simulate cells with an increased intrinsic binding rate (*k*_on0_). In the model, bulky glycoproteins are treated as a continuous, elastic barrier rather than discrete entities with defined surface density; their presence is reflected through mechanical properties (thickness and stiffness) that contribute to the energy barrier Δ*E*_bg_. Reducing MUC1 levels effectively lowers Δ*E*_bg_, thereby shifting the cells into a different adhesion state. To preserve the formulation $k_{\mathrm{on}}=k_{\mathrm{on}0}\exp\left(-\frac{\Delta E}{k_{B}T}\right)$, we mathematically incorporated the reduction in Δ*E*_bg_ from the MUC1 knockdown into an effective increase in *k*_on0_, in order to investigate model predictions regarding the context‐dependent role of MUC15. Experimental characterizations of focal adhesions in modified and unmodified pancreatic cancer cell lines verified predictions of the existence of the three predicted adhesion states, and demonstrated transitions between them (Figure 5j; Table S1, Supporting Information). Specifically, MUC15 overexpression in MiaPaca‐2 cells corresponded to state (ii) (low *k*_on0_; high *p*_sg_), yielding inhibited integrin clustering and small/immature focal adhesions; MUC1 knockdown corresponded to state (iii) (high *k*_on0_; high *p*_sg_), resulting in random integrin binding and weak focal adhesions.

Overexpressing MUC15 in MiaPaca‐2 cells switched the adhesion state from (***i***) to a (***ii***) by inhibiting integrin clustering. Increasing the intrinsic integrin binding rate in MiaPaca‐2 cells by knocking down the bulky glycoprotein MUC1 (Figure S12, Supporting Information) switched the adhesion state from (***i***) to (***iii***) by attenuating the “kinetic trap” effect (Figure 5j; Figure S13, Supporting Information). This observation is consistent with prevailing views on the role of a bulky glycocalyx in breast ^[^ ^2^ ^]^ and prostate cancer,^[^ ^12^ ^]^ in which depletion of bulky glycoprotein (e.g., MUC1) inhibits focal adhesion growth and thus carcinogenesis. Finally, overexpressing MUC15 in MUC1‐depleted cells switched the adhesion state from cluster‐less state (***iii***) back to the clustering state (***i***) by recovering the energy gradient of integrin binding, thus facilitating focal adhesion assembly (Figure 5j; Figure S13, Supporting Information). Overall, by varying the expression levels of MUC15 (a smaller‐sized glycoprotein) and MUC1 (a larger‐sized glycoprotein), we validated the predicted changes in focal adhesion patterns. These results reinforce the role of glycoprotein size and composition in dynamically regulating focal adhesion assembly and adhesion state transitions.

To investigate whether knocking down MUC15 influences the composition and structure of bulky glycocalyx, we conducted a systematic analysis of major glycocalyx components. Western blot analysis revealed that modulating MUC15 levels did not significantly alter the expression of other major mucins (MUC1, MUC4, and MUC16) in either Panc‐1 or MiaPaca‐2 cells (Figure S14, Supporting Information). We further examined potential effects on overall glycocalyx structure using succinylated wheat germ agglutinin (s‐WGA) lectin staining.^[^ ^40^, ^41^, ^42^ ^]^ While MUC1 knockdown, used as a positive control, substantially reduced glycosylation levels, MUC15 overexpression in both control and MUC1 knockdown groups did not significantly alter glycosylation patterns (Figure S15, Supporting Information). These results support our hypothesis that small glycoproteins like MUC15 are independent of the bulky glycocalyx.

Notably, although MiaPaca‐2 cells express low levels of some mucins compared to other PDAC lines, we found that even these lower expression levels could be manipulated effectively. Specifically, we achieved significant MUC1 knockdown in MiaPaca‐2 cells at both mRNA and protein levels (Figure S12, Supporting Information), supporting their utility for studying glycocalyx‐dependent processes.

### 2.7 Ectodomain Truncation Confirms the Critical role of MUC15 Size in Determining its Function

To directly assess whether MUC15's functional effects are determined by its ectodomain size, we conducted a systematic domain truncation analysis. We generated three distinct MUC15 variants in MUC15‐deficient MiaPaca‐2 cells and comprehensively evaluated their effects on cell migration and focal adhesion assembly (Figure S16, Supporting Information). These variants included: 1) full‐length MUC15 containing the complete protein sequence, 2) an ectodomain‐truncated construct (MUC15‐ΔTR) lacking the extracellular domain from the first N‐glycosylation site to the last O‐glycosylation site while preserving the transmembrane and cytoplasmic domains, and 3) a cytoplasmic‐tail‐deleted variant (MUC15‐ΔCT) maintaining the complete extracellular domain but lacking the intracellular signaling region (Figure S16a, Supporting Information).

This experimental design specifically isolated the contribution of ectodomain size from potential signaling effects, allowing us to determine whether MUC15's influence on cellular behavior derived primarily from physical or biochemical mechanisms. Cell migration analysis revealed a clear ectodomain size‐dependent pattern. Expression of the ectodomain‐truncated construct (MUC15‐ΔTR) failed to significantly alter cell migration speed compared to control cells (Figure S16b,c, Supporting Information), demonstrating that the mere presence of MUC15's cytoplasmic domain was insufficient to influence motility. In contrast, both full‐length MUC15 and the cytoplasmic‐tail‐deleted variant (MUC15‐ΔCT) significantly inhibited migration (Figure S16b,c, Supporting Information), providing compelling evidence that the physical presence of the ectodomain, rather than intracellular signaling, drives MUC15's functional effects.

Focal adhesion analysis further reinforced this size‐dependent mechanism. Quantification of focal adhesion dimensions revealed that cells expressing either full‐length MUC15 or MUC15‐ΔCT developed significantly smaller focal adhesions compared to control cells, while MUC15‐ΔTR‐expressing cells showed no significant alterations in focal adhesion morphology (Figure S16d,e, Supporting Information). The parallel effects observed in both migration and focal adhesion assembly provide strong evidence that these cellular behaviors are regulated by the physical dimensions of MUC15's extracellular domain rather than through cytoplasmic signaling cascades.

Together, these domain truncation experiments establish that the mechanical properties conferred by MUC15's extracellular domain size, rather than its biochemical signaling capacity, are the primary determinants of its effects on cell migration and focal adhesion assembly. This mechanistic insight explains how MUC15, despite being a relatively small glycoprotein compared to mucins like MUC1, can significantly influence cancer cell behavior through size‐dependent physical interactions at the cell‐matrix interface.

### 2.8 MUC15 Overexpression Reduces Tumor Metastasis and ECM Remodeling In Vivo

**Figure 6:**
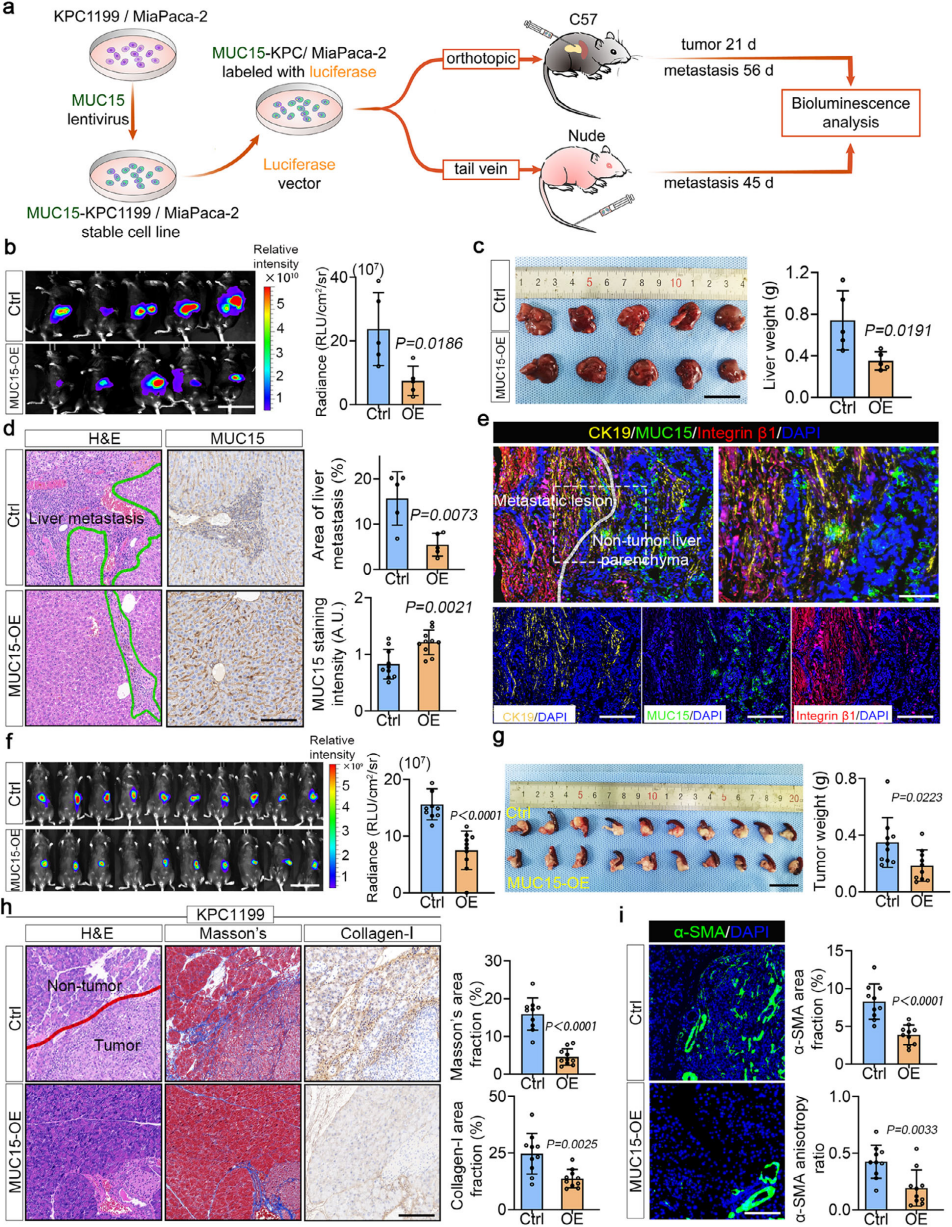
MUC15 overexpression reduces tumor metastasis and ECM remodeling in vivo. a) Schematic overview of the experimental design for syngeneic orthotopic transplantation and tail vein injection models to study metastatic progression. b) Bioluminescence imaging of metastatic burden in the orthotopic model using luciferase‐labeled KPC1199 cells expressing either control vector or MUC15 (left) with quantification of bioluminescence signal intensity (right). *n* = 5 mice per group. c) Representative gross pathology of hepatic metastases from the orthotopic model (left) with corresponding liver weight quantification (right). *n* = 5 mice per group. d) H&E staining of hepatic metastatic lesions (outlined in green) and MUC15 immunostaining (left) with quantification of metastatic area and MUC15 staining intensity (right). *n* = 5 mice per group. e) Multiplex immunofluorescence analysis of non‐tumor liver parenchyma and intrahepatic metastatic foci showing spatial distribution of MUC15 (green), CK19 (yellow), integrin β1 (red), and nuclei (DAPI, blue). f) In vivo bioluminescence imaging of primary tumor growth in the orthotopic model (left) with quantification of signal intensity (right). *n* = 10 mice per group. g) Gross pathology of primary pancreatic tumors (left) with tumor weight quantification (right). *n* = 10 mice per group. h) Analysis of stromal remodeling by H&E, Masson's trichrome, and collagen immunofluorescence staining (left) with quantification of fibrillar collagen coverage area (right). i) α‐SMA immunostaining of tumor sections (left) with quantification of α‐SMA‐positive area coverage and α‐SMA anisotropy ratio (right). *n* = 10 mice per group. Statistical analyses were performed using an unpaired two‐tailed Student's *t*‐test. Scale bars: 10 mm (a, c), 5 mm (g), and 100 µm (d, e, h, i).

Because shifting amongst these three adhesion states can change focal adhesions from conditions known to promote metastasis and tumor development to conditions known to attenuate these, we next tested whether switching the adhesion state of cells could affect these factors in vivo. To characterize MUC15's role in metastasis within the pancreatic tumor microenvironment, we developed two complementary in vivo models. Our primary model utilized orthotopic transplantation of syngeneic KPC1199 cells in C57BL/6 mice, which preserves the unique characteristics of the pancreatic tumor microenvironment. We complemented this with tail vein injection of MiaPaca‐2 cells in SCID mice as a secondary model (**Figure** **6**a). In both models, we compared the metastatic behavior of MUC15‐overexpressing cells (adhesion state ii) with control cells (adhesion state i). The orthotopic model revealed that tumors derived from MUC15‐overexpressing cells showed significantly reduced metastatic burden in liver (Figure 6b–d), with parallel results observed in the tail vein model (Figure S17a,b, Supporting Information). To characterize MUC15's role in metastatic colonization, we performed immunohistochemical analysis of liver sections containing metastatic lesions. We used CK19 as a PDAC‐specific marker to identify tumor cells within the liver tissue. Our analysis revealed elevated MUC15 expression in the liver tissue adjacent to metastatic sites, while the metastatic lesions themselves showed strong staining for both CK19 and integrin β1(Figure 6e). This distinct staining pattern supports our hypothesis that MUC15 acts as an inhibitor of liver metastasis. These results verified the prediction that MUC15 inhibits tumor metastasis in vivo, consistent with our observations in human tissues (Figure 1).

Within the pancreatic tumor microenvironment, cancer‐associated fibroblasts (CAFs) play a crucial role in ECM remodeling and cancer cell invasion.^[^ ^43^, ^44^, ^45^, ^46^ ^]^ Since our data showed that MUC15 regulates YAP (Figure 2), and because YAP's downstream target CTGF is a key activator of CAFs, we investigated this signaling axis. MUC15 depletion in Panc‐1 cells increased CTGF secretion, while MUC15 overexpression in MiaPaCa‐2 cells reduced it (Figure S18, Supporting Information). These findings were supported by changes in CTGF gene expression (Figure 2h). The physiological relevance of these changes was evident in human PDAC tissues, which showed denser, more organized collagen networks compared to normal tissues (Figure S17c, Supporting Information).

To further characterize microenvironmental interactions, we examined how MUC15‐mediated changes in YAP activity affect pancreatic stellate cells (PSCs). Co‐culture experiments revealed that MUC15‐depleted Panc‐1 cells induced higher α‐SMA expression in PSCs compared to control cells (Figures S19 and S20, Supporting Information), indicating enhanced PSC activation. In our orthotopic model, MUC15 overexpression significantly reduced tumor growth (Figure 6f,g) and ECM remodeling, as evidenced by Masson's staining and collagen fiber analysis (Figure 6h). Similar effects were observed in the subcutaneous injection model (Figure S17d, Supporting Information). Importantly, tumors from MUC15‐overexpressing cells showed decreased YAP nuclear localization (Figure S17d,e, Supporting Information) and reduced α‐SMA expression (Figure 6i; Figure S17e, Supporting Information), indicating that MUC15 regulates both direct metastasis and microenvironment remodeling through tumor cell‐PSC interactions.

## 3 Discussion

Our results demonstrate that cells can adopt three distinct adhesion states, influenced by glycocalyx ectodomain size and density, which govern MUC15's functional effects on integrin dynamics and focal adhesion formation. Multiple lines of experimental evidence establish that ectodomain size, rather than biochemical signaling, determines glycoprotein function. First, comparative analyses revealed that MUC15 (334 amino acids) localizes within focal adhesions and directly interacts with integrin β1, while larger glycoproteins like MUC1 (>1000 amino acids) are excluded from these structures. Second, ectodomain truncation experiments demonstrated that removing MUC15's extracellular domain eliminated its inhibitory effects on cell migration and focal adhesion assembly, while preserving this domain, even without the cytoplasmic signaling tail, maintained full functionality. Third, manipulating the relative proportions of differently sized glycoproteins shifted cells between adhesion states with distinct integrin clustering patterns, as predicted by our mathematical model. Finally, these size‐dependent effects translated to in vivo outcomes, where MUC15 overexpression reduced metastasis and ECM remodeling in pancreatic cancer models. Together, these findings establish a mechanical basis for glycoprotein function in cancer progression, wherein the physical dimensions of the ectodomain, rather than its biochemical properties, dictate cellular adhesion dynamics and subsequent mechanotransduction signaling.

In our model, bulky and small glycoproteins differ in two aspects: ectodomain length and membrane mobility. Bulky glycoproteins are treated as immobile, consistent with Paszek's lattice‐spring framework,^[^ ^2^, ^3^ ^]^ while small glycoproteins are allowed to diffuse laterally within the membrane. Other mechanical properties, such as molecular stiffness, are held constant. This simplification was made to isolate the effect of size and diffusion behavior on integrin clustering. Although real glycoproteins may differ in additional properties, such as stiffness, such parameters are difficult to measure experimentally. Therefore, we used equivalent stiffness values (i.e., σ_sg_ = σ_bg_) as a first‐order approximation. While this does not capture the full molecular complexity, the combined differences in length and mobility are sufficient to produce distinct adhesion regimes. The model is thus intended to offer qualitative insight into size‐dependent adhesion transitions, rather than quantitatively precise predictions.

Integrated modeling and experiment show that these effects arise through integrin activation and the integrin‐YAP mechanotransduction axis. The kinetic trap model,^[^ ^3^ ^]^ which underlies the prevailing understanding that increased expression of glycocalyx molecules monolithically promotes cancer growth, survival and metastasis,^[^ ^2^, ^47^ ^]^ represents only one of the three possible states, a state that is achieved when the glycocalyx is dominated by larger (>1000 amino acid) mucins such as MUC1,^[^ ^48^ ^]^ MUC4,^[^ ^8^ ^]^ and MUC16,^[^ ^48^ ^]^ or by a relatively uniform distribution of smaller mucins. Intermediate levels of smaller (<500 amino acids) glycoproteins such as MUC15 ^[^ ^17^, ^21^ ^]^ or MUC14 ^[^ ^49^ ^]^ can switch a cell to a different adhesion state so as to inhibit cancer development. Results explain the multifaceted role of the glycocalyx in cancer progression and highlight a critical role for the specific molecular composition of the glycocalyx.

Our study also sheds light on how MUC15 impacts the microenvironment of tumors through effects on ECM remodeling and PSCs activation. Depending on the adhesion state, depletion of MUC15 can activate PSCs through paracrine signaling mediated by YAP‐induced cytokine secretion. Similarly, manipulating cells by overexpressing MUC15 in MiaPaca‐2 cells inhibited both integrin‐mediated focal adhesion assembly and the ECM remodeling in vivo by tumor cell‐activated PSCs, resulting in suppressed tumor metastasis and progression. Thus, MUC15 influences not only tumor cell behavior but also stromal cell regulation and ECM remodeling. These alterations affect both the integrin‐YAP mechanotransduction axis and ECM remodeling, and have the potential to further inhibit tumor metastasis and progression. Understanding the mechanisms underlying these observations could provide insights into therapeutic strategies targeting the tumor microenvironment and ECM remodeling.

While our study focuses on the modulation of single‐cell integrin‐ECM interactions by glycocalyx architecture, we acknowledge that metastatic dissemination often occurs through collective migration, which involves both cell‐ECM and cell‐cell adhesion,^[^ ^50^ ^]^ particularly during epithelial‐to‐mesenchymal transition (EMT).^[^ ^51^ ^]^ In this context, glycoproteins may also regulate cadherin‐mediated intercellular adhesions and thereby influence collective invasion. For example, overexpression or depletion of the glycoprotein podocalyxin has been shown to modulate E‐cadherin levels in a context‐dependent manner, with consequences for tumor growth and metastasis.^[^ ^22^ ^]^ Moreover, glycoprotein CD44 mediates adhesion between tumor cells and endothelial cells by binding to deposited hyaluronic acid, thereby facilitating the trans‐endothelial migration and invasion.^[^ ^52^ ^]^ The size‐dependent steric effects of glycoproteins proposed in our study may also influence cadherin‐based intercellular adhesions and, by extension, collective migration dynamics. Although these mechanisms lie beyond the scope of our current model, they represent important directions for future investigation into how glycocalyx composition governs not only single‐cell but also multicellular behaviors during cancer progression.

Targeting the glycocalyx and its interactions with both integrins and the tumor microenvironment has been proposed as a chemotherapeutic strategy, but has yet to be successful.^[^ ^4^, ^22^, ^47^, ^50^, ^51^, ^52^ ^]^ Results suggest mechanisms underlying the multifaceted role of glycocalyx molecules in promoting or attenuating cancer progression, and identify conditions in which targeting the glycocalyx may prove effective. Glycocalyx composition may thus prove promising for both diagnosis and therapy.

## 4 Experimental Section

### Ethics Approval and Consent to Participate

Female athymic BALB/c nu/nu mice received a standard chow diet freely and were housed in conditions of 12:12 h dark: light cycle, 22 ± 1 °C ambient temperature, and 50 ± 10% humidity. All the mice were reared in a specific pathogen‐free (SPF) professional chamber at the Experimental Animal Center of Xi'an Jiaotong University, Xi'an, China. All animal experiments were performed in accordance with a protocol approved by the Department of Laboratory Animal Center, Xi'an Jiaotong University Health Science and supervised by the institutional review board of Xi'an Jiaotong University. All studies of human patient samples were approved by the Research Ethics Committee of the First Affiliated Hospital of Xi'an Jiaotong University.

### Cell Culture

Human PDAC cell lines Panc‐1(RRID:CVCL_0480), BxPc‐3 (RRID:CVCL_0186) and MiaPaCa‐2 (RRID:CVCL_0428) were purchased from the Chinese Academy of Sciences Cell Bank of Type Culture Collection (CBTCCCAS, Shanghai, China). Cells used in this study were confirmed to be free of mycoplasma contamination and were passaged in the laboratory for less than 6 months after receipt. Panc‐1, BxPc‐3, and MiaPaca‐2 cells were cultured in DMEM (Gibco, Grand Island, NY, USA) supplemented with 10% FBS (HyClone, Logan, UT, USA) and 1% penicillin‐streptomycin (Gibco) under 5% CO2 and 37 °C. All cell lines were grown on fibronectin‐coated 6‐well plates (140 675, Thermo Fisher Scientific). The rationale for using specific cell lines in this study is stated in the experimental results section.

Human pancreatic stellate cells (PSCs) were originally collected from patient tissue. Pancreatic adjacent tissue (1.0–1.5 g) was obtained from patients undergoing a pancreatic cancer partial resection at the Department of Hepatobiliary Surgery of The First Affiliated Hospital of Xi'an Jiaotong University and was immediately placed in sterile ice‐cold Hanks balanced salt solution (HBSS) containing 100 U mL^−1^ penicillin and 100 µg mL^−1^ streptomycin (Gibco). Histological diagnostic assessment of specimens was confirmed by pathologists. Human PSCs were isolated using a density gradient method, as previously described.^[^ ^53^ ^]^ Isolated PSCs were maintained at 37 °C with 5% CO_2_ in DMEM/F12 (HyClone, Logan, USA) supplemented with 10% heat‐inactivated fetal bovine serum (FBS) (HyClone), 100 U mL^−1^ penicillin, and 100 µg mL^−1^ streptomycin. PSCs were identified by oil red staining of intracellular fat droplets and immunofluorescence of α‐smooth muscle actin (α‐SMA) immunofluorescence.

### Plasmid and Lentiviral Transfections

Short hairpin RNA (shRNA) was used to knock down MUC15 in Panc‐1 cells. LVRU6GP plasmids that contained the shRNA of MUC15 were purchased from GeneCopoeia (Guangzhou, China), and a pLKO.1 lentiviral system (Oligoengine, Seattle, WA) was constructed to infect cells. For the overexpression of MUC15, the lentiviral system EX‐E2664‐Lv201 (GeneCopoeia, Guangzhou, China) containing full‐length MUC15 was used. Lentiviruses were produced in HEK293T cells by co‐transfection with the pCMV‐VSV‐G (RRID: Addgene_8454) and psPAX2 (RRID: Addgene_12 260) vectors using Lipofectamine 2000 according to the manufacturer's instructions. The medium was changed to DMEM, high glucose supplemented with 10% FBS after 12 h, and the viral particles were collected after an additional 24 h. The viral supernatants were centrifuged and sterile‐filtered before being mixed 1:1 in cancer cell culture medium. After 24 h of transfection with 8 µg mL^−1^ polybrene, following the manufacturer's instructions, fresh culture medium supplemented with 1 µg mL^−1^ puromycin was added to the cells. Successful transduction was verified using confocal microscopy and Western blotting.

### Transwell Migration and Invasion Assays

Cell migration and invasion were quantified using Transwell chambers with 8 µm pores (Millipore, Boston, USA). For invasion assays, the upper chamber was coated with 60 µL of Matrigel (BD Biosciences, USA, diluted 1:5 in serum‐free medium) and allowed to polymerize at 37 °C for 4 h. For migration assays, chambers were left uncoated. Cells (8× 10^4^) in 300 µL of serum‐free medium were added to the upper chambers, with complete medium containing 20% FBS in the lower chamber as a chemoattractant. After 24 h or 48 h of incubation at 37 °C, non‐migrated cells were removed from the upper surface with a cotton swab. Migrated cells were fixed with 4% paraformaldehyde, stained with 0.1% crystal violet, and counted in five random fields per membrane under a 20× objective using light microscopy (Leica).

### Wound Healing Assay

Cells were cultured to 90% confluence in 6‐well plates, and then wounded using a sterile 200 µL pipette tip to create a uniform scratch. Debris was removed by washing twice with PBS, and fresh serum‐containing medium was added. Wound closure was monitored by phase‐contrast microscopy, with images captured at 0‐, 24‐, and 48‐h post‐wounding using an inverted light microscope (Leica) at 100× magnification. At least four random fields per condition were photographed, with wound areas measured using ImageJ. The percentage of wound closure was calculated as: $\frac{\mathrm{Initial}\;\mathrm{wound}\;\mathrm{area}-\mathrm{Final}\;\mathrm{wound}\;\mathrm{area}}{\mathrm{Initial}\;\mathrm{wound}\;\mathrm{area}}\times 100\%$.

### EdU Proliferation Assay

Cell proliferation was measured using the 594 Click‐iT EdU Imaging Kits (Yeasen, catalog # 40276ES60). Cells were seeded at 5 × 10^4^ cells well^−1^ in 24‐well plates and cultured for 12 h before adding EdU (10 µM final concentration). After 3 h of EdU incorporation at 37 °C, cells were fixed with 4% paraformaldehyde for 10–15 min at room temperature and permeabilized with 0.1% Triton X‐100 for 10 min. The click chemistry reaction was performed according to the manufacturer's instructions, incubating cells with the reaction cocktail for 30 min at room temperature in the dark. After washing with PBS, nuclei were counterstained with DAPI (1 µg mL^−1^). Images were acquired using a fluorescence microscope (Carl Zeiss) with a 20× objective. The percentage of EdU‐positive cells was calculated from at least 300 cells per condition across three independent experiments.

### ORIS Migration Assay

Panc‐1 and MiaPaca‐2 cells expressing MUC15‐GFP were plated at a concentration of 2×10^5^ cells per well on fibronectin (5 µg mL^−1^; Sigma–Aldrich, F1141) coated 96‐well plates fitted with stoppers (Platypus Technologies, CMA1.101, Lot#: 20H0503). Cells were incubated overnight in a humidity chamber at 37 °C and 5% CO_2_ before the stoppers were removed, and then incubated for an additional 24 h. Fluorescence images were taken using the low light microscope system (×4 and ×5 magnification) at 0, 12, and 24 h upon removal of stoppers, and cell migration was quantified.

### Preparation of PEG Hydrogels and the Mechanical Characterization of PEG Hydrogels

PEG hydrogels were synthesized as described previously.^[^ ^29^ ^]^ An ADHR3 shear rheometer (TA Instruments) with a parallel plate geometry (8 mm in diameter) was used for rheological testing. The storage modulus, G′, and loss modulus, G′′, were measured at 1% strain and a frequency of 1 rad s^−1^. The 14 mm diameter PEG hydrogel samples were tested at a constant temperature of 37 °C. Effective Young's modulus *E* was calculated as follows:

(1) $$E=2\left(1+\nu \right)\sqrt{{G^{\prime }}^{2}+{G^{\prime \prime }}^{2}}$$

where ν  =  0.5 is Poisson's ratio of the PEG hydrogels.^[^ ^54^, ^55^ ^]^

### Immunohistochemistry

4‐mm sample sections were incubated with anti‐MUC15 (1:500, HPA026110, Sigma–Aldrich), Anti‐act‐integrin β1 (1:200, MAB2079Z, MilliporeSigma), anti‐integrin β1 (1:200, ab30394, abcam), and anti‐YAP (1:400, 14 074, CST), respectively, overnight at 4 °C in a humidified chamber, followed by incubation with the horseradish peroxidase‐conjugated secondary antibodies at 37 °C for 30 min. Staining was completed by 1–2 min of incubation with diaminobenzidine substrate, which results in a brown‐colored precipitate at the antigen site. The tissue sections stained immunohistochemically were analyzed, and the mean staining intensity was calculated using Image‐Pro Plus software.

### Real‐Time Quantitative PCR

Extraction of total RNA from tumor cells was achieved using a Fastgen 200 RNA isolation system (Fastagen Biotechnology, Shanghai, China). RNA samples were transcribed reversely by PrimeScript RT Master Mix (Takara Bio, Dalian, China). The amplifications were run using a QuantStudio 7 real‐time PCR system (Applied Biosystems), with SYBR Green master mix (Takara Bio, Dalian, China).

### Immunoblotting

Western blotting was performed according to standard procedures. The cells lysates were prepared using RIPA buffer (50 mM Tris, pH 8.0, 150 mM NaCl, 0.1% SDS, 1% NP40 and 0.5% sodium deoxycholate), containing proteinase inhibitors (1% inhibitors cocktail and 1 mM PMSF) (Roche Applied Science, Germany) for 10 min on ice and centrifuged at 13000g for 15 min at 4 °C. The samples were boiled, and resolved in 4–20% Tris‐glycine gels (Invitrogen) and subsequently transferred to polyvinylidene fluoride (PVDF) membranes. The PVDF membranes were blocked with 5% bovine serum albumin in Tris‐buffered saline with 0.02% Tween20 (TBS‐T) for 1 h, probed with primary antibodies diluted in 3% BSA in TBS‐T overnight at 4 °C, and subsequently with secondary antibody conjugated to horseradish peroxidase diluted in 5% milk in TBS‐T for 1–2 h at room temperature. After washing the membranes three times with TBST buffer. Visualization was performed by using an ECL chemiluminescent detection system (Bio‐rad, USA). A list of all the antibodies used in this study, including their working dilutions, can be found in Table S2 (Supporting Information). All data represent at least three independent experiments.

### Immunostaining

Collected samples were fixed at desired time points, using 4% paraformaldehyde for 20 min at room temperature. Samples were rinsed thrice with PBS, then permeabilized with 0.3% Triton X‐100 for 15 min. Non‐specific binding sites were subsequently blocked with 5% bovine serum albumin (BSA) for 45 min at room temperature. Samples were then incubated with the primary antibody diluted in 1% BSA overnight at 4 °C. After three PBS rinses, samples were incubated with fluorescently labeled secondary antibodies for 1.5 h at room temperature, followed by F‐actin staining using Rhodamine Phalloidin (CST, 8953) for a 30 min incubation. All immunostaining samples were embedded in ProLong Gold Antifade Reagent with DAPI (Invitrogen, Lot: P36982) and visualized with an Olympus FV3000 confocal microscope.

### Traction Force Microscopy

8‐arm PEG maleimide was doped with 0.2 µm diameter fluorescent microspheres at 2% v/v (Invitrogen, F8811), and then mixed with 8‐arm PEG thiol to form hydrogels. Human Panc‐1 and MiaPaca‐2 cells were cultured on the surface of the PEG hydrogels for 24 h before traction force microscopy (TFM) analysis was performed. Phase contrast images of a single cell and fluorescence images of the embedded fluorescent microspheres were captured on an Olympus FV3000 confocal microscope. Image sequences for each cell were taken before and after cell lysis with SDS (sodium dodecyl sulfate) buffer. All imaging was performed in an environmental chamber (37 °C, 5% CO_2_). By comparing bead positions with and without cells, a map of gel deformations caused by cells was obtained using custom particle‐imaging‐velocimetry software. Then, assuming that gel displacements were caused by forces exerted by cells in the cell‐gel contact area, the corresponding map of cell forces was calculated using a previously described algorithm.^[^ ^56^ ^]^ The average forces exerted by each cell were then calculated.

### Quantification of YAP nuc/cyto Ratio

For the YAP nucleus‐to‐cytoplasm (nuc/cyto) ratios, the nucleus and cytoplasm were identified by F‐actin staining and DAPI staining, respectively. The YAP nuc/cyto ratio, *R*, was calculated following a previously reported procedure,^[^ ^25^, ^57^, ^58^ ^]^ in which the ratio of the total fluorescence intensity in the nucleus, *I*_nucleus_, to the total fluorescence in the remainder of the cell, was weighted by the areas of the nucleus and the remainder of the cell:

(2) $$R=\frac{\left(I_{\mathrm{nucleus}}/A_{\mathrm{nucleus}}\right)\;}{\left(I_{\mathrm{cell}}-I_{\mathrm{nucleus}}\right)/\left(A_{\mathrm{cell}}-A_{\mathrm{nucleus}}\right)}$$

where *A*_nucleus_ is the area of the nucleus as measured by DAPI staining, *A*_cell_ is the overall area of the cell as delineated by F‐actin staining, and *I*_cell_ is the total fluorescence intensity in the overall cell. Intensities and areas were measured using Image J.

### Quantification of F‐Actin Anisotropy

Actin anisotropy calculations were carried out using a freely‐available plugin for ImageJ, FibrilTool, as previously described.^[^ ^59^ ^]^ Briefly, F‐actin images of the cell were taken, and the inner region of the cell was selected as the quantification area. Anisotropy ratios were reported on a scale from 0 (perfectly isotropic) to 1 (perfectly anisotropic).

### Enzyme‐Linked Immunosorbent Assay (ELISA)

Cells on fibronectin‐coated plates were conditioned in serum‐free medium for 48 h. The culture media were collected and centrifuged at 1000 × g for 20 min at 4 °C to remove particles, and the supernatants were frozen at −80 °C until use. The production of CTGF in the supernatants of PSCs was assessed by ELISA using a commercially available ELISA kit (Elabscience, Lot: E‐EL‐H0828) according to the manufacturer's instructions.

### Co‐Immunoprecipitation

Co‐immunoprecipitation assay was performed by using an immunoprecipitation kit from Abcam (ab206996, Cambridge, UK) following the manufacturer's protocol. Briefly, Panc‐1 and MiaPaca‐2 cells plated on 10‐cm plates were lysed in immunoprecipitation buffer (150 mM NaCl, 10 mM Tris–HCl (pH 7.4), 1 mM EDTA, 1 mM EGTA (pH 8.0), 0.2 mM sodium orthovanadate, 0.2 mM PMSF, 1% Triton X‐100, and 0.5% NP‐40) and protease inhibitors (Roche). The lysates were incubated overnight with 2 µg anti‐MUC15, integrin β1 antibody at 4 °C, followed by the addition of 30 µL Protein A/G agarose and a gentle vortex and incubation for 1 h at 4 °C according to the manufacturer's instructions. The beads were collected by magnetic force and washed three times with 1 mL immunoprecipitation buffer. The beads were re‐suspended in 30–50 µL 2 × laemmli buffer with 100 mM dithiothreitol. The immunoprecipitates were analyzed by SDS‐PAGE.

### Flow Cytometry Assays

Cells were trypsinized using EDTA‐free trypsin and immediately re‐suspended in PBS containing 1% BSA at 1 × 10^6^ cells mL^−1^. Cell aliquots (100 µL) were incubated with Human FcR block (Invitrogen, Lot: 00‐4409‐42) on ice for 30 min (Human FcR block was used to block non‐specific staining of fluorescent antibody FC receptors with purified antibodies specifically directed against Fc receptors). After washing twice with 1% BSA in PBS, the cell pellet was re‐suspended in a staining solution of AlexaFluor‐conjugated primary antibodies (1:300; ThermoFisher) in PBS with 1% BSA. Cells were washed twice with PBS (1% BSA) and re‐suspended in PBS (1% BSA). Twenty thousand events were analyzed by FACSverse (BD Biosciences), and further analyses of the collected data points were performed using FlowJo.

### Generation of Stable Cell Lines Expressing Different Forms of MUC15

To systematically investigate size‐dependent effects, stable cell lines expressing three distinct MUC15 variants were generated using lentiviral transduction as previously described.^[^ ^2^ ^]^ Starting with the MUC15 splice variant (NM145650), protein sequences were designed based on detailed analysis of MUC15's glycosylation profile. The variants were 1) full‐length MUC15 (complete protein sequence); 2) extracellular‐truncated construct (MUC15‐ΔTR), created by removing the extracellular domain from the first N‐glycosylation site (position 25) to the last O‐glycosylation site (position 238), while preserving the transmembrane and cytoplasmic domains; and 3) cytoplasmic‐tail‐deleted construct (MUC15‐ΔCT), generated by removing the C‐terminal segment (residues 261–334) while maintaining the complete extracellular domain. This design strategy enabled us to directly compare the effects of extracellular domain size versus cytoplasmic signaling on cellular behavior. These constructs were synthesized by GeneCopoeia (Guangzhou, China) as lentiviral vectors containing:

**Table:** 

Full length MUC15	h‐MUC15(NM_145650)‐pLV‐CMV‐MCS‐EF1‐ZsGreen1‐T2A‐Puro
MUC15‐ΔTR	h‐MUC15(ΔTR)‐pLV‐CMV‐MCS‐EF1‐ZsGreen1‐T2A‐Puro
MUC15‐ΔCT	h‐MUC1S(ΔCT)‐pLV‐CMV‐MCS‐EF1‐ZsGreen1‐T2A‐Puro

### Animal Experiments

All animal experiments were conducted following institutional guidelines and were approved by the local ethics committee at the Department of Laboratory Animal Center, Xi'an Jiaotong University Health Science. Female athymic BALB/c nu/nu mice ≈4–6 weeks old were used for the experiment.

For the tail‐vein injection model of cancer metastasis, ten mice per group were randomly distributed into two groups (MUC15‐OE and Ctrl), 2×10^6^ cells were suspended in 200 µL PBS, and injected via the tail vein. ≈6–8 weeks after injection, D‐luciferin substrate (Biosynth, Naperville, IL, USA) in PBS with 450 mg kg^−1^ was injected into the peritoneal cavity; 15 min later, mice were anesthetized and bioluminescence imaging (BLI) was performed to detect the distant metastases in the lung and other organs.

For the subcutaneous xenotransplanted tumor model, ten mice per group were randomly distributed into two groups. The cells were re‐suspended in a 1:1 (v/v) mixture of culture medium and Matrigel (BD Biosciences, USA), and 0.8 × 10^6^ MiaPaca‐2‐MUC15‐OE cells + 0.2 × 10^6^ PSCs, 0.8 × 10^6^ MiaPaca‐2‐MUC15 Ctrl cells + 0.2 × 10^6^ PSCs, were injected into the right flank of nude mice. After 6–8 weeks, the mice were sacrificed, and the xenograft tumors were harvested, weighed, and processed for immunohistochemistry staining.

### PDAC Metastasis Model

Both the murine KPC1199 cell line was used (a gift from Professor Jing Xue, State Key Laboratory of Oncogenes and Related Genes, Shanghai Cancer Institute, Ren Ji Hospital, School of Medicine, Shanghai Jiao Tong University ^[^ ^60^, ^61^ ^]^) and the human PDAC cell line MiaPaCa‐2. The KPC1199 line, derived from KPC mice, was selected for the syngeneic orthotopic model, while MiaPaCa‐2 cells were used for the tail vein injection model. Stable cell lines were first established by transducing both KPC1199 and MiaPaCa‐2 cells with lentiviral vectors expressing either control or MUC15‐overexpressing constructs. These cells were then further modified with a luciferase reporter vector to enable in vivo bioluminescence imaging of tumor progression.

### Orthotopic Transplantation Model

KPC1199 cells were cultured in DMEM supplemented with 10% fetal bovine serum, 1% penicillin‐streptomycin, and 1% glutamine. During the logarithmic growth phase, cells were harvested, washed with PBS, and resuspended in a 1:5 mixture of PBS and Matrigel at a concentration of 4 × 10^4^ cells µL^−1^. Female C57BL/6J mice (6–8 weeks old) were anesthetized with isoflurane, and a small left abdominal incision was made to expose the pancreas. The cell suspension (25 µL) was injected into the pancreatic tail. After confirming the absence of leakage, the pancreas was returned to the abdominal cavity, and the incision was closed with sutures and surgical clips. Mice were monitored postoperatively for signs of distress or complications. Tumor growth was tracked weekly using ultrasound or palpation, and at experimental endpoints, the primary tumor and major organs were collected for histological analysis. To characterize MUC15's role in metastatic colonization, immunohistochemical analysis of liver sections containing metastatic lesions was performed. CK19 was used as a PDAC‐specific marker to identify tumor cells within the liver tissue.

### Statistical Analysis

Statistical comparisons were performed with GraphPad Prism version 7.0 software (GraphPad Software, USA) using one‐way analysis of variance (ANOVA) with Tukey's post hoc test for comparison of multiple groups, and using Student's *t*‐test for comparison between two groups. The threshold for statistically significant differences between groups was *p* < 0.05. In all figures, data were shown as mean ± standard error (s.e.m.) unless otherwise stated. All experiments were repeated independently at least thrice, except where noted. The number of cells counted for each condition was indicated in each figure legend.

## Conflict of Interest

The authors declare no conflict of interest.

## Author Contributions

S.Z. and H.Z. contributed equally to this work. M.L., Z.W., S.M.Z., and H.Y.Z. designed the study, in consultation with G.M.G., T.J.L., F.X., and S.M.Z. H.Y.Z., Z.E.Z., S.W., Y.Q.S., J.W., X.L.C., J.P.L., W.K.Q., Y.Y.Y., Q.H.X., and Z.P.R. performed the experiments, modeling, collected, and analyzed the data. M.L., Z.W., S.M.Z., H.Y.Z., G.M.G., T.J.L., Q.L., Y.M.Z., and F.X. prepared the manuscript. All authors discussed the experiments, modeling, read, and commented on the manuscript.

## Supporting information



Supporting Information

## Data Availability

Data sharing is not applicable to this article as no new data were created or analyzed in this study.
