# Supplementary material for: MUC15 Ectodomain Architecture Regulates Integrin Clustering to Control Cancer Metastasis
Source: Adv Sci (Weinh). 2025 Oct 13;12(48):e13552. doi: 10.1002/advs.202513552 (PMC12752613; doi:10.1002/advs.202513552)
Supplement: Supplementary file 1 — Supporting Information [file ADVS-12-e13552-s001.docx]

Supplementary Materials for

**“****MUC15 Ectodomain Architecture Regulates Integrin Clustering to Control Cancer Metastasis”**

Simei Zhang^1,2,3*^, Hongyuan Zhu^4,5*^, Zeen Zhu^1,2^, Shuai Wu^1,2^, Yiqun Song^1,2^, Jin Wang^4,5^, Xinlong Chen^1,2^, Weikun Qian^1,2^, Jianpeng Li^6^, Yangyang Yue^7^, Qinhong Xu^2,8^, Zhiping Ruan^9^, Qing Li^3^, Yaomin Zhu^3^, Tian Jian Lu^10^, Guy M. Genin^4,5,11,12^, Feng Xu^4,5^, Zheng Wang^1,2#^, Min Lin^4,5#^

1 Department of Hepatobiliary Surgery, The First Affiliated Hospital of Xi'an Jiaotong University, Xi'an 710061, P.R. China

2 Pancreatic Disease Treatment Center of Xi’an Jiaotong University, Xi’an 710061, P.R. China

3 Department of Anesthesiology & Center for Brain Science, The First Affiliated Hospital of Xi’an Jiaotong University, Xi'an 710061, P.R. China

4 The Key Laboratory of Biomedical Information Engineering of Ministry of Education, School of Life Science and Technology, Xi’an Jiaotong University, Xi’an 710049, P.R. China

5 Bioinspired Engineering and Biomechanics Center (BEBC), Xi’an Jiaotong University, Xi’an 710049, P.R. China

6 Department of Cardiovascular Surgery, The First Affiliated Hospital of Xi’an Jiaotong University, Xi'an 710061, P.R. China

7 Department of Vascular Surgery, The First Affiliated Hospital of Xi’an Jiaotong University, Xi'an 710061, P.R. China

8 Department of Geriatric Surgery, The First Affiliated Hospital of Xi’an Jiaotong University, Xi’an 710061, P.R. China

9 Department of Medical Oncology, The First Affiliated Hospital of Xi'an Jiaotong University, Xi’an 710061, P.R. China

10 State Key Laboratory of Mechanics and Control of Mechanical Structures, Nanjing University of Aeronautics and Astronautics, Nanjing 210016, P.R. China

11 Department of Mechanical Engineering & Materials Science, Washington University in St. Louis, St. Louis 63130, MO, USA

12 NSF Science and Technology Center for Engineering Mechanobiology, Washington University in St. Louis, St. Louis 63130, MO, USA

* These authors contributed equally to this work

# Corresponding authors: minlin@xjtu.edu.cn; zheng.wang11@mail.xjtu.edu.cn

**The PDF file includes:**

**Supplementary Note. Hierarchical, coarse-grained model of how the glycocalyx affects integrin dynamics**

**Supplementary Figures**

Supplementary Fig. 1 mRNA expression levels of MUC15 collected from public database and PDAC cell lines.

Supplementary Fig. 2 MUC15 knockdown effciency and cell morphology.

Supplementary Fig. 3 MUC15 overexpression effciency and cell morphology.

Supplementary Fig. 4 Impact of MUC15 expression level on cancer cell migration and invasion.

Supplementary Fig. 5 Assessment of cell proliferation using EdU assay.

Supplementary Fig. 6 MUC15 inhibits YAP nuclear translocation through direct mechanotransduction.

Supplementary Fig. 7 Localization of MUC15 with key focal adhesion components.

Supplementary Fig. 8 Interaction between MUC15 and integrin β1 in Panc-1 and MiaPaca-2 cells.

Supplementary Fig. 9 Fabrication and Young’s moduli of PEG hydrogels

Supplementary Fig. 10 Focal adhesion counting analysis confirms MUC15's effects on integrin β1 activation.

Supplementary Fig. 11 An illustration labeling the various length scales used for calculating integrin binding.

Supplementary Fig. 12 Characterization of MUC1 manipulation in MiaPaca-2 cells

Supplementary Fig. 13 Quantification of focal adhesion lengths in cells with different expression levels of glycoproteins (related to Fig. 5j).

Supplementary Fig. 14 The impact of MUC15 on composition and glycosylation of the glycocalyx.

Supplementary Fig. 15 Assessment of global glycocalyx structure using s-WGA lectin staining in MiaPaca-2 cells.

Supplementary Fig. 16 Direct demonstration that MUC15's extracellular domain size controls cell migration and focal adhesion assembly.

Supplementary Fig. 17 Controlling the cell adhesion state by modulating MUC15 expression controls cancer metastasis and ECM remodeling in a mouse model of PDAC.

Supplementary Fig. 18 MUC15 regulates CTGF secretion.

Supplementary Fig. 19 Assessment of pancreatic stellate cell (PSC) activation.

Supplementary Fig. 20 MUC15 dependent paracrine effect on PSC activation.

**Supplementary Tables**

Table S1: Mapping of adhesion states to model and experiments

Table S2: Reagent or resource.

Table S3: List of input parameter values.

**Supplementary Reference**

Supplementary Note. Hierarchical, coarse-grained model of how the glycocalyx affects integrin dynamics

A mathematical model was established to identify why the cancer-promoting role of large glycoproteins ^1, 2^, known to arise through kinetic trap-driven integrin clustering, is not consistently observed in small glycoproteins. We developed a hierarchical, coarse-grained model of integrin dynamics that extends the model of Paszek, et al. ^1, 3^, to consider the effects of two distinct sets of glycocalyx components, differing in size.

**Spatial representation**

The cell-ECM interface was represented using a lattice spring model, with the cell membrane and substrate each comprising a flat, parallel spring network ($100\times100\times3$ nodes for membrane, and $100\times100\times21$ nodes for substrate, lattice spacing 10 nm). The total number of nodes on the membrane surface was $n_{\mathrm{node}}=$ $100\times10$0. Glycoproteins were separated into two components: bulky glycoproteins that spanned the distance between the cell membrane and substrate, and small glycoproteins randomly distributed on the cell membrane, each modeled as an independent linear spring. Integrins ($n_{\mathrm{int}}$ in number) were dispersed randomly over the membrane and could either bind to substrate or diffuse freely on the membrane. The proportion of small glycoproteins occupying membrane nodes was $p_{\mathrm{sg}}=\frac{n_{\mathrm{sg}}}{n_{\mathrm{node}}}, where n_{\mathrm{sg}}$ represents the number of small glycoproteins, was prescribed for each simulation.

**Integrin reactions**

Within the model, integrin exhibits three different conformational states (*i.e.* inactive, active and bound). Among these states, only the active integrin is capable of binding to ligands. These states undergo reversible transformations through two sets of reversible chemical reactions ^3^:

| $I_{\mathrm{inact}} \begin{matrix} \underset{\to}{k_{\mathrm{act}}} \\ \overset{\leftarrow}{k_{\mathrm{deact}}} \end{matrix}I_{\mathrm{act}}\begin{matrix} \underset{\to}{k_{\mathrm{on}}} \\ \overset{\leftarrow}{k_{\mathrm{off}}} \end{matrix}I_{\mathrm{bin}}$ | (3) |
| --- | --- |

where $I_{\mathrm{inact}}$, $I_{\mathrm{act}}$ and $I_{\mathrm{bin}}$ represent the populations of inactive, active and bound integrins, respectively. The rates of activation ($k_{\mathrm{act}}$) and deactivation ($k_{\mathrm{deact}}$) determine the dynamic shift between active and inactive states. Similarly, binding ($k_{\mathrm{on}}$) and unbinding ($k_{\mathrm{off}}$) rates govern the interaction of integrins with ligands respectively. $k_{\mathrm{act}}$ and $k_{\mathrm{deact}}$ are influenced by factors including divalent cations, cell signaling, and adaptor proteins.

Consider an active integrin located near a bound integrin and preparing to bind to the substrate (**Supplementary Fig. 11**). The binding rates ($k_{\mathrm{on}}$) between the active integrin and its nearest ligand is calculated according to ^3^:

| $k_{\mathrm{on}}=k_{on0}\exp\left( -\frac{\Delta E}{k_{B}T} \right)$ | (4) |
| --- | --- |

where $k_{on0}$ represents the intrinsic binding rate, and $\Delta E$ is the minimum elastic energy which changes with bond formation, and $k_{B}$ reflects the thermal energy. The energy change ($\Delta E$) is composed of three components:

| $\Delta E=\Delta E_{0}+{\Delta E}_{b}+{\Delta E}_{g}$ | (5) |
| --- | --- |

where $\Delta E_{0}$ is the elastic energy change of the membrane and substrate, while ${\Delta E}_{b}$ is accounts for the elastic energy change of integrin-ligand bond. ${\Delta E}_{g}$ incorporates the elastic energy change of glycoproteins. When substrate is significantly stiffer than the cell membrane, $\Delta E_{0}$ can be simplified as:

| $\Delta E_{0}=\frac{1}{2}\sigma_{m}\left( l_{0}-l_{b} \right)^{2}$ | (6) |
| --- | --- |

where $\sigma_{m}$ represents the spring constant of cell membrane, $l_{0}$ denotes the distance between the membrane and the substrate before integrin-ligand bond formation, $l_{b}$ is equilibrium length of integrin-ligand bond without force. ${\Delta E}_{g}$ includes two parts and is denoted as:

| ${\Delta E}_{g}={\Delta E}_{\mathrm{bg}}+{\Delta E}_{\mathrm{sg}}$ | (7) |
| --- | --- |

where ${\Delta E}_{\mathrm{bg}}$ is the elastic energy change of bulky glycoproteins, while ${\Delta E}_{\mathrm{sg}}$ is the elastic energy change of small glycoproteins. ${\Delta E}_{\mathrm{bg}}$ and ${\Delta E}_{\mathrm{sg}}$ are expressed as ^4^:

| ${\Delta E}_{\mathrm{bg}}=\frac{1}{2}\sigma_{\mathrm{bg}}{(l_{\mathrm{bg}}-l_{b})}^{2}-\frac{1}{2}\sigma_{\mathrm{bg}}{(l_{\mathrm{bg}}-l_{0})}^{2}$ | (8) |
| --- | --- |
| ${\Delta E}_{\mathrm{sg}}=\left\{ \begin{aligned} \frac{1}{2}\sigma_{\mathrm{sg}}\left( l_{\mathrm{sg}}-l_{b} \right)^{2} l_{0}\geq l_{\mathrm{sg}} \\ \frac{1}{2}\sigma_{\mathrm{sg}}\left( l_{\mathrm{sg}}-l_{b} \right)^{2}-\frac{1}{2}\sigma_{\mathrm{sg}}\left( l_{\mathrm{sg}}-l_{0} \right)^{2} l_{0}<l_{\mathrm{sg}} \end{aligned} \right.$ | (9) |

where $\sigma_{\mathrm{bg}}$ is the spring constant of bulky glycoprotein layer, $\sigma_{\mathrm{sg}}$ is the spring constant of small glycoprotein layer, $l_{\mathrm{bg}}$ is the equilibrium thickness of bulky glycoprotein layer without deformation, $l_{\mathrm{sg}}$ is the equilibrium length of small glycoprotein layer without deformation.

The unbinding rate ($k_{\mathrm{off}}$) of bound integrins depends on the stretching force in the integrin-ligand bond, according to Bell model ^5^:

| $k_{\mathrm{off}}=k_{off0}\exp\left( F_{b}/F_{0} \right)$ | (10) |
| --- | --- |

where $k_{off0}$ represents the intrinsic unbinding rate, $F_{b}$ denotes the force on integrin-ligand bond determined by the force balance between membrane, integrin-ligand bond and substrate, and $F_{0}$ is the characteristic rupture force of integrin-ligand bond.

**Diffusion of membrane proteins**

In the model, bulky glycoproteins are treated as immobile, *i.e.*, without diffusion, consistent with Paszek’s lattice-spring framework ^1, 3^. In contrast, small glycoproteins are allowed to diffuse laterally within the membrane with a diffusion coefficient of $D_{\mathrm{sg}}={10}^{2} \mathrm{nm}^{2}/s$, which is much slower than that of integrins ($D_{\mathrm{int}}={10}^{4} \mathrm{nm}^{2}/s$). The hopping rate $k_{\mathrm{diff}}$ for integrins and small glycoproteins between adjacent lattice points is calculated as follows ^3^:

| $k_{\mathrm{diff}}=\frac{4D}{\Delta l^{2}}$ | (11) |
| --- | --- |

where $D$ signifies the diffusion coefficient of integrins or small glycoprotein within the membrane, and $\Delta l$ represents the step length of the hop, which is equal to lattice spacing.

**Mechanical equilibrium on cell-ECM interface**

Following each integrin binding or unbinding event, the force equilibrium between adjacent nodes on the membrane and substrate was recalculated, ensuring that the vector sum of forces on each node equals zero:

| $\boldsymbol{F}_{i}=-\sum_{j} \sigma_{ij}\left( \frac{\left\vert r_{ij} \right\vert-l_{ij}}{\left\vert r_{ij} \right\vert} \right)\boldsymbol{r}_{ij}=0$ | (12) |
| --- | --- |

where $\left| r_{ij} \right|$ denotes the distance between nodes *i* and *j*, and $\sigma_{ij}$ and $l_{ij}$ respectively represent the spring constant and equilibrium length of the spring connecting nodes *i* and *j*.

Boundary nodes at the substrate base remained fixed in position, whereas the boundary nodes at the cell membrane base moved to balance forces. Boundary nodes on the membrane surface experienced forces from integrin-ligand bonds, bulky glycoproteins and small glycoproteins. Forces from integrin-ligand bonds on the membrane and substrate surfaces were calculated by:

| $F_{b}=\sigma_{b}\left\vert l-l_{b} \right\vert$ | (13) |
| --- | --- |

where $\sigma_{b}$ is the spring constant of integrin-ligand bond, $l$ is the distance between the membrane and substrate at the bonded site, and $l_{b}$ is the equilibrium length of unforced integrin-ligand bond.

Forces from the overall bulky glycoprotein layer on membrane and substrate surface were calculated as:

| $F_{\mathrm{bg}}=\sigma_{\mathrm{bg}}\left\vert l-l_{\mathrm{bg}} \right\vert$ | (14) |
| --- | --- |

Forces from small glycoproteins on the membrane and substrate surfaces were calculated as:

| $F_{\mathrm{sg}}=\sigma_{\mathrm{sg}}\left\vert l-l_{\mathrm{sg}} \right\vert$ | (15) |
| --- | --- |

**Data analysis**

To analyze integrin clustering, we employed Ripley’s K-function to integrin membrane positions projected onto the x-y plane, as previously reported ^3^. First, the K-function of integrin distribution is calculated as:

| $K(s)=\frac{1}{A}\sum_{i}^{n_{\mathrm{int}}} \sum_{j}^{n_{\mathrm{int}}} W_{ij}\left( s \right)$ | (16) |
| --- | --- |

where$i$ and $j$ represent summations over all integrin positions, $A$ is the area of the cell membrane projected onto the *x*-*y* plane, $s$ represents the sampling radius, and $W_{ij}\left( s \right)$ equals one if the distance between points $i$ and $j$ is less than $s$, and zero otherwise. Periodic boundaries were applied when calculating $W_{ij}\left( s \right)$. Subsequently, $K(s)$ was transformed as follows:

| $R\left( s \right)=\sqrt{\frac{K\left( s \right)}{s}}-s$ | (17) |
| --- | --- |

For a completely random point pattern, the expected value of $R\left( s \right)$ is zero, while for a clustered points pattern, $R\left( s \right)$ is positive. $R\left( s \right)$ was calculated over a continuous range of sampling radii, and the maximal value of $R\left( s \right)$ ($R_{\max}$) was set as the integrin clustering index.

Supplementary Figures

### Supplementary Figure 1


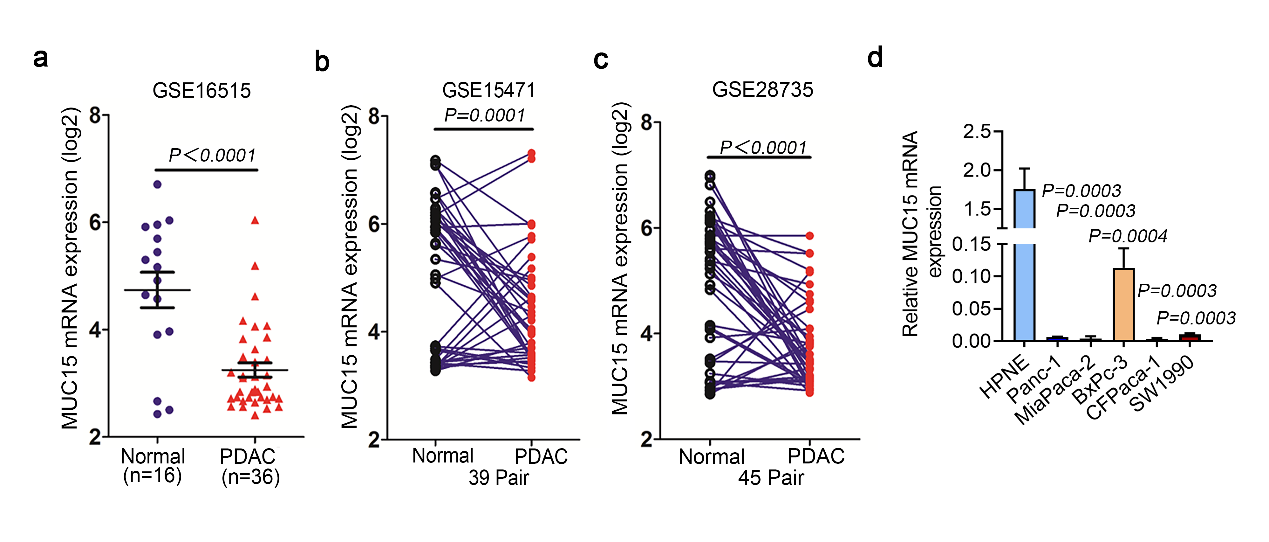


**Supplementary Fig. 1 mRNA expression levels of MUC15 collected from public database and PDAC cell lines**. **a-c,** Downregulated mRNA expression levels of MUC15 in PDAC in datasets of GSE16515 (*n* = 16/36), GSE15471 (*n* = 39 pair) and GSE28735 (*n* = 45 pair). **d,** mRNA expression levels of MUC15 in the normal pancreatic duct epithelium cell line (HPNE) and PDAC cell lines (Panc-1, MiaPaca-2, BxPc-3, CFPaca-1 and SW1990). *P* values were obtained using unpaired two-tailed Student’s t-test (a) and paired Student’s t-test (b, c) one-way ANOVA test (d).

### Supplementary Figure 2

**
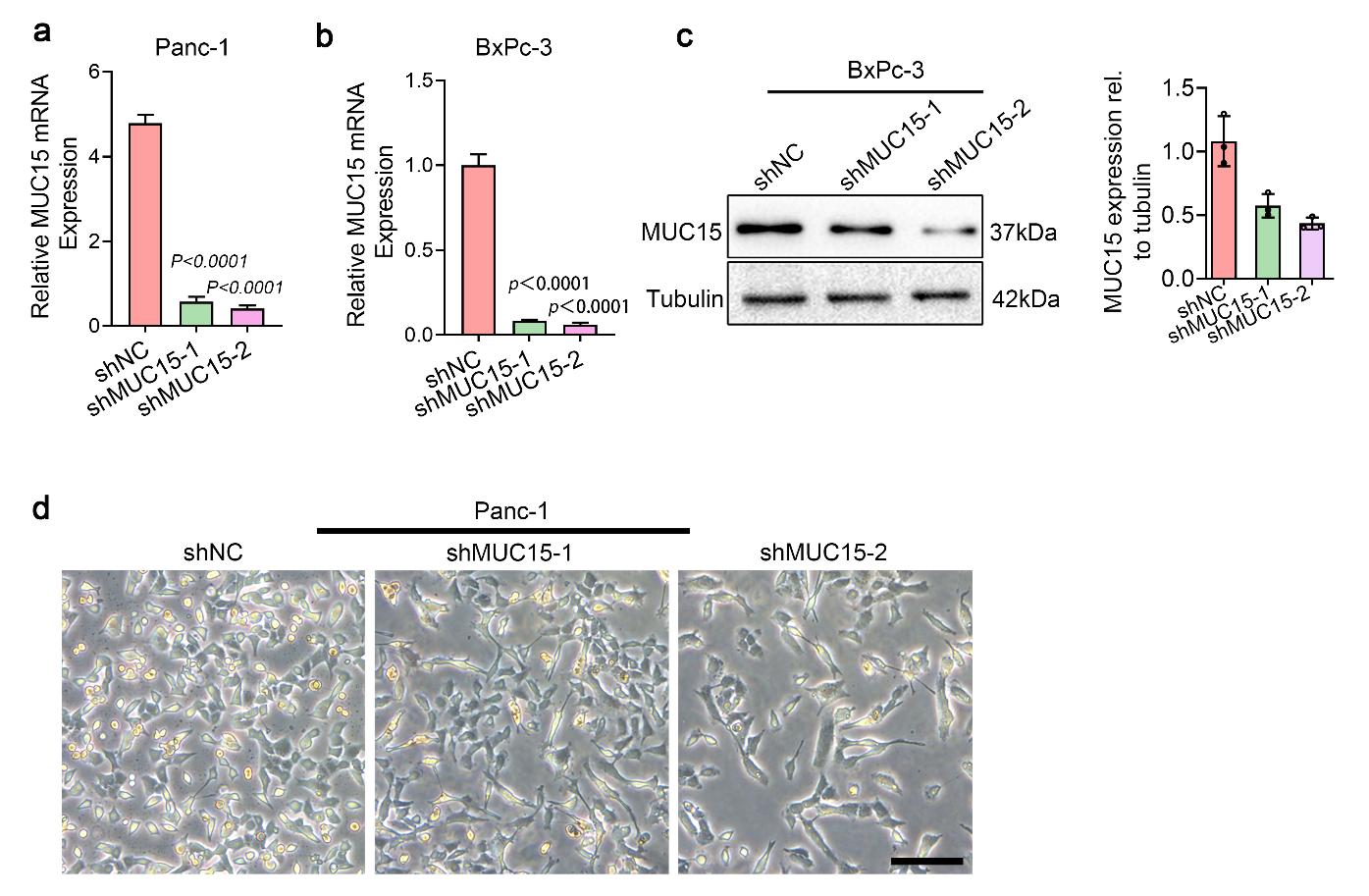
**

**Supplementary Fig. 2** **MUC15 knockdown effciency and cell morphology**. **a, b,** Quantitative real-time RT-PCR analysis of MUC15 knockdown efficiency in Panc-1 and BxPc-3 cell lines, respectively. The endogenous control used for quantitative real-time RT-PCR was 18S (*n* = 3). *P* values were obtained using one-way ANOVA. **c**, Western blotting analysis of the MUC15 knockdown efficiency in BxPc-3 cell line. Tublin was used as a loading control (*n* = 3). **d**, MUC15-depleted Panc-1 cells exhibited an elongated morphology and the formation of thin membrane protrusions. Scale bar: 100 μm.

### Supplementary Figure 3


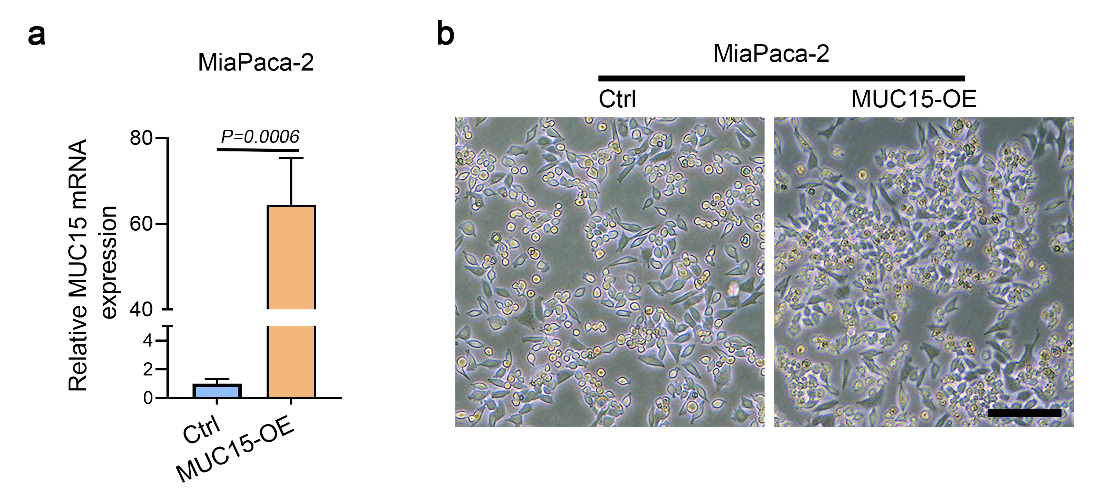


**Supplementary Fig. 3** **MUC15 overexpression alters gene expression and cell morphology. a**, MUC15 mRNA levels in MiaPaca-2 cells measured by quantitative RT-PCR, normalized to 18S rRNA (*n* = 3 independent experiments). Statistical analysis by unpaired two-tailed Student's t-test. **b**, Phase contrast microscopy showing morphological changes in MUC15-overexpressing MiaPaca-2 cells, including cell rounding and reduced protrusion formation. Scale bar: 100 μm.

### Supplementary Figure 4


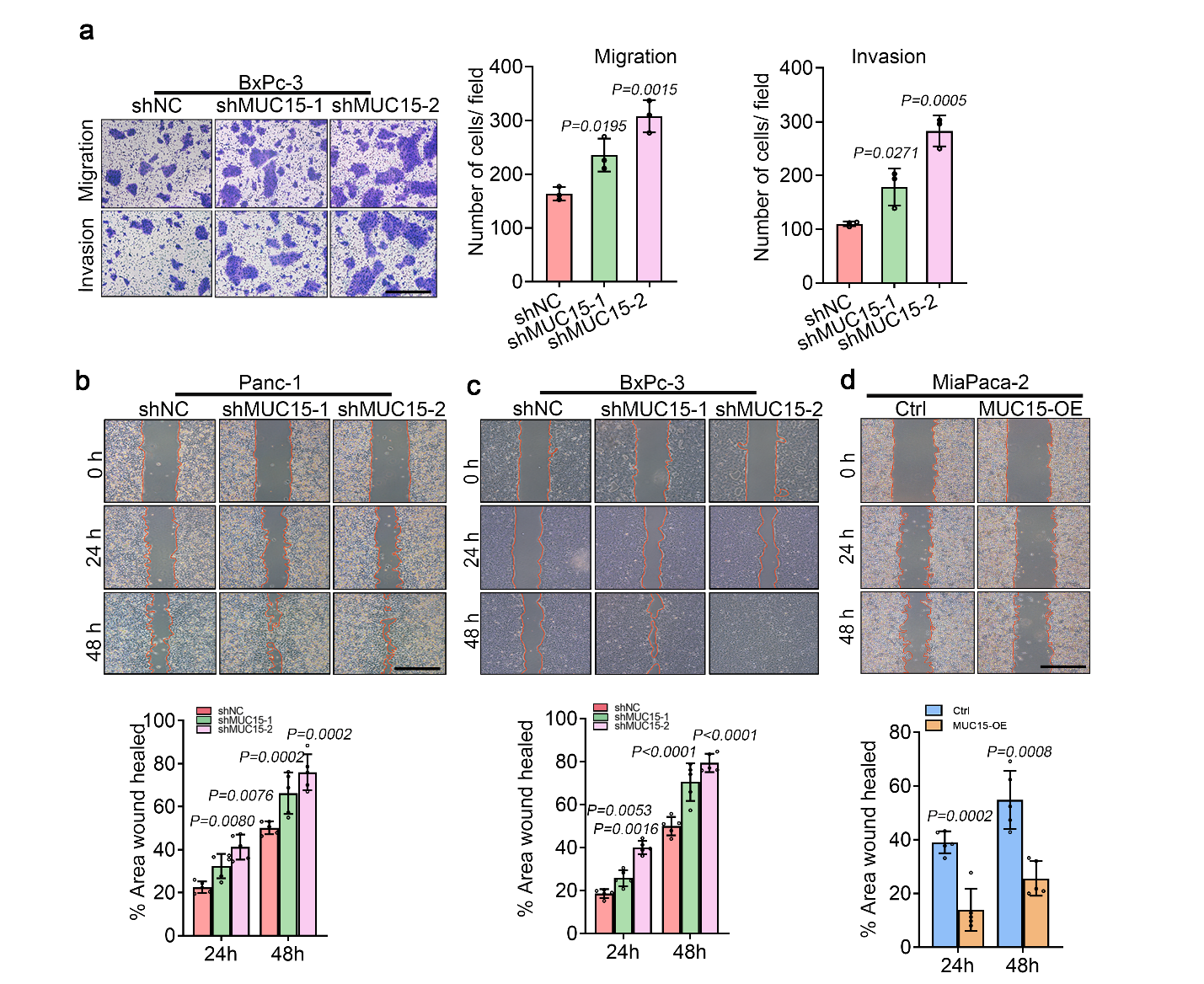


**Supplementary** **Fig. 4** **Impact of MUC15 expression level on cancer cell migration and invasion**. **a**, Left: Representative images of Matrigel transwell migration and invasion assays in MUC15-depleted BxPc-3 cells. Right: Corresponding quantification of number of cells per field. **b**-**d**, Top: Representative images of wound healing assays in MUC15-depleted cells (Panc-1 and BxPc-3) and MUC15-overexpressing MiaPaCa-2 cells, respectively. Dashed lines indicate the initial gap at the beginning of the assay. Bottom: Corresponding quantification of wound gap area relative to 0 h. The experiments were independently repeated three times. Data are presented as mean ± s.e.m.. *P* values were obtained using one-way (**a**-**c**) ANOVA and unpaired two-tailed Student’s t-test (**d**). Scale bar: 100 μm (**a**-**d**).

### Supplementary Figure 5


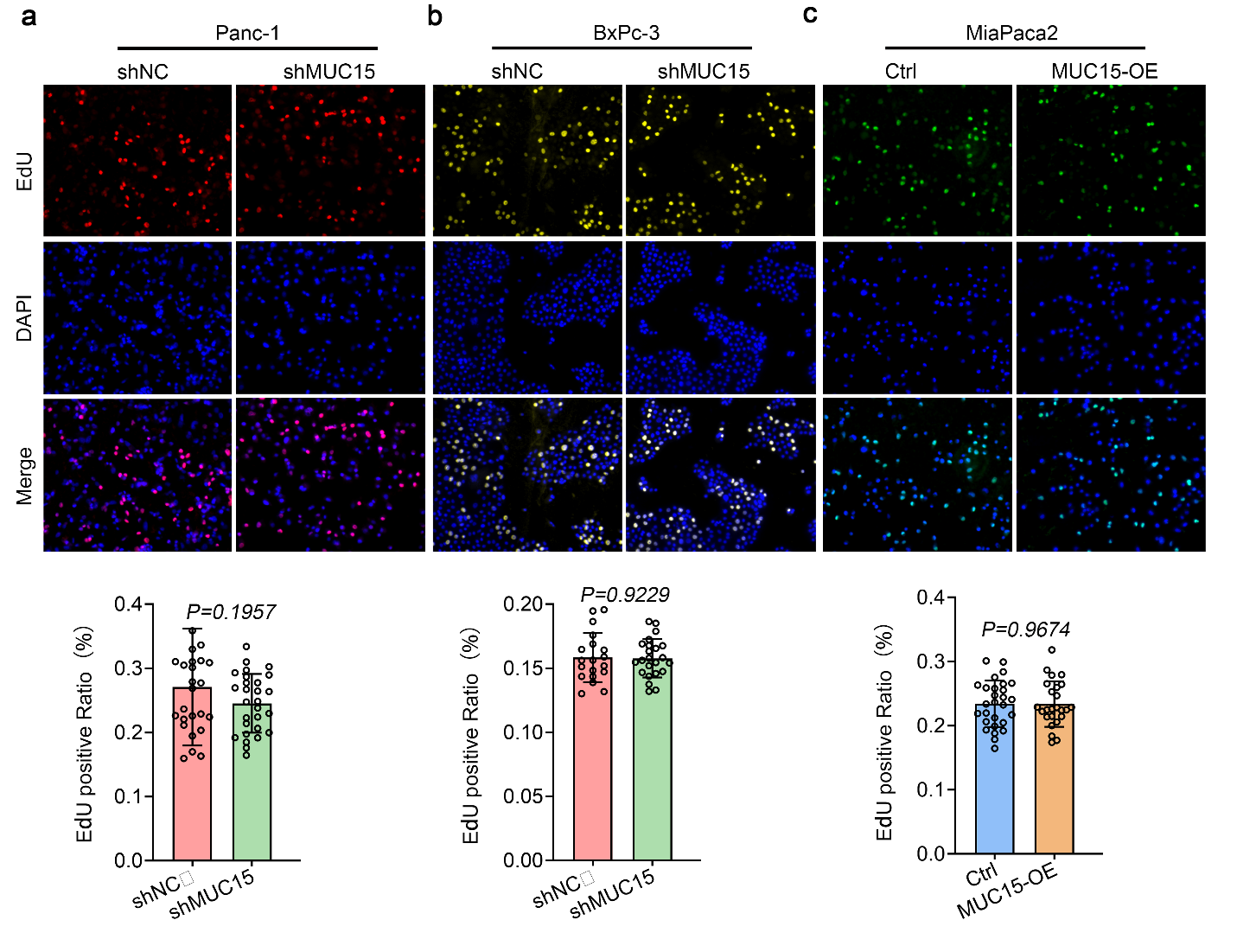


**Supplementary Fig. 5** **Assessment of cell proliferation using EdU assay.** **a**-**c**, Cell proliferation in MUC15-depleted Panc-1 and BxPc-3 cells, as well we MUC15-overexpressing MiaPaca-2 cells. **Top：**Representative images of EdU staining in MUC15-depleted Panc-1 and BxPc-3 cells and MUC15-overexpressing MiaPaca-2 cells cultured for 2 day. **Bottom:** Corresponding quantification of the ratio of EdU-positive cells per field. The results indicate that alteration of MUC15 expression levels does not affect cell proliferative capacity. Data are presented as mean ± s.e.m., and *P* values were obtained using unpaired two-tailed Student’s t-test.

### Supplementary Figure 6


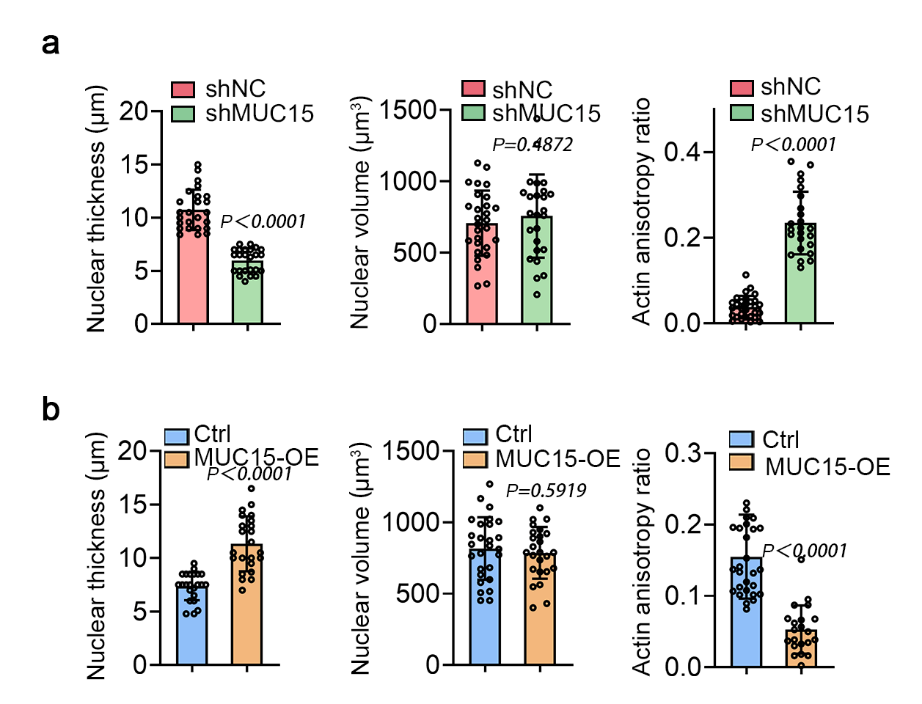


**Supplementary Fig. 6 MUC15 inhibits YAP nuclear translocation through direct mechanotransduction. a,** Nuclear thickness (*n* = 25, 24 cells), nuclear volume (*n* = 28, 25 cells) and actin anisotropy ratio (*n* = 29, 24 cells) for conditions in F**igure 2h**. **b**, Nuclear thickness (*n* = 23, 23 cells), nuclear volume (n = 27, 25 cells) and actin anisotropy ratio (*n* = 28, 21 cells) for conditions in panel F**igure 2i**. Statistical analyses: unpaired two-tailed Student's t-test.

### Supplementary Figure 7


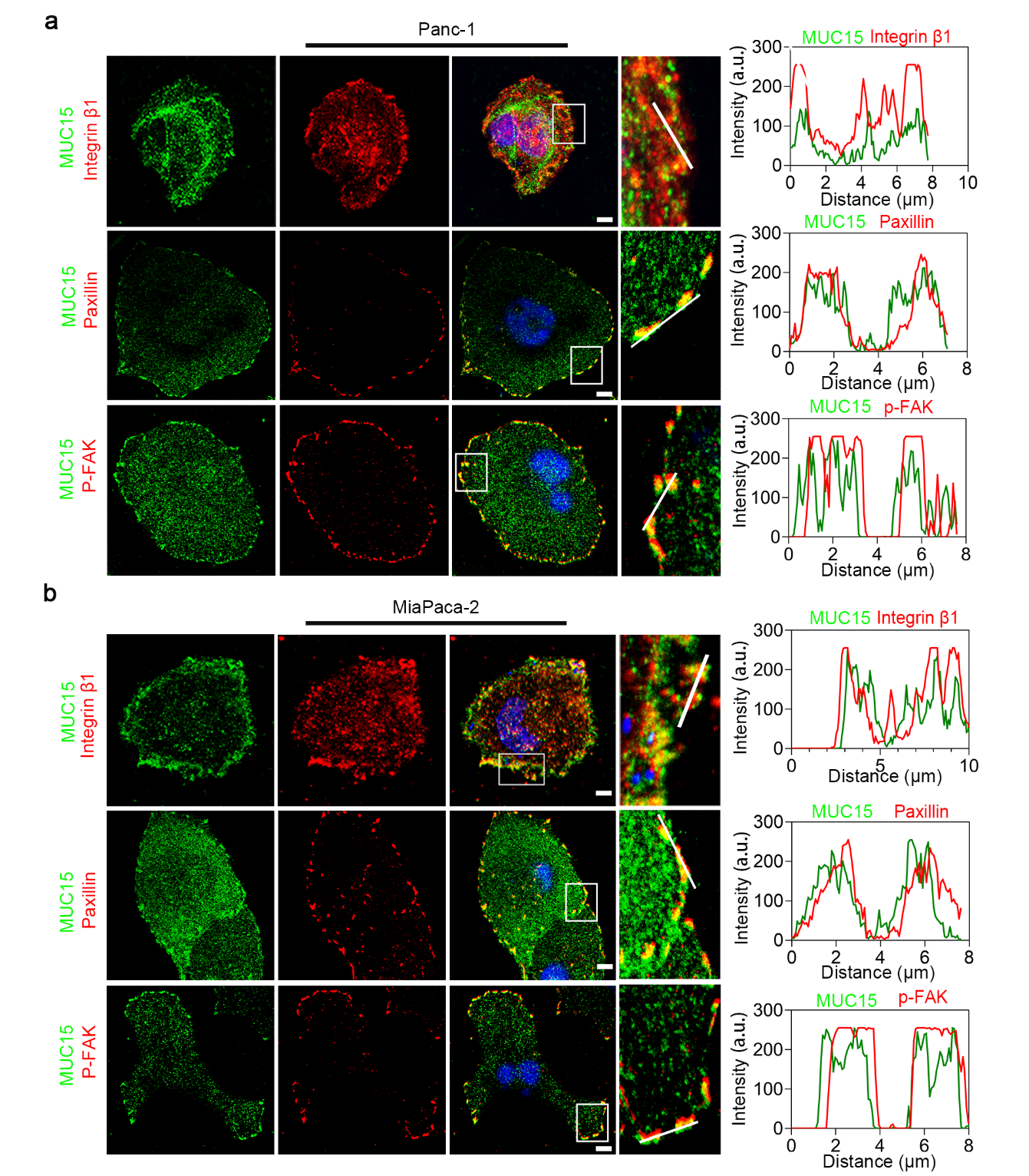


**Supplementary Fig. 7** **Localization of MUC15 with key focal adhesion components**. **a, b,** **Left**: Immunostaining of MUC15, integrin β1, paxillin and p-FAK in Panc-1 and MiaPaca-2 cells. **Right**: Corresponding co-location analysis of MUC15 with paxillin, integrin β1 and p-FAK, respectively. Results indicate the localization of MUC15 at focal adhesions. Scale bars: 20 μm.

### Supplementary Figure 8


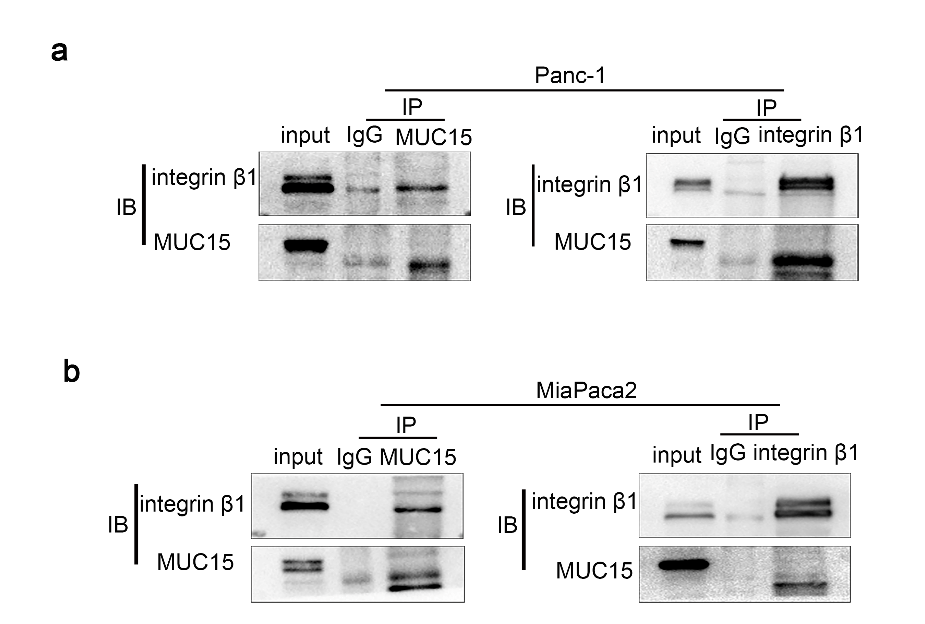


**Supplementary Fig**. **8 Interaction between MUC15 and integrin β1 in Panc-1 and MiaPaca-2 cells.** **a, b,** Co-immunoprecipitation (IP) was performed using antibodies against MUC15 or integrin β1, followed by immunoblot detection of the respective proteins. MUC15 was found to pulls down integrin β1, and vice versa, in Panc-1 cells (**a**) and MiaPaca-2 cells (**b**).

### Supplementary Figure 9


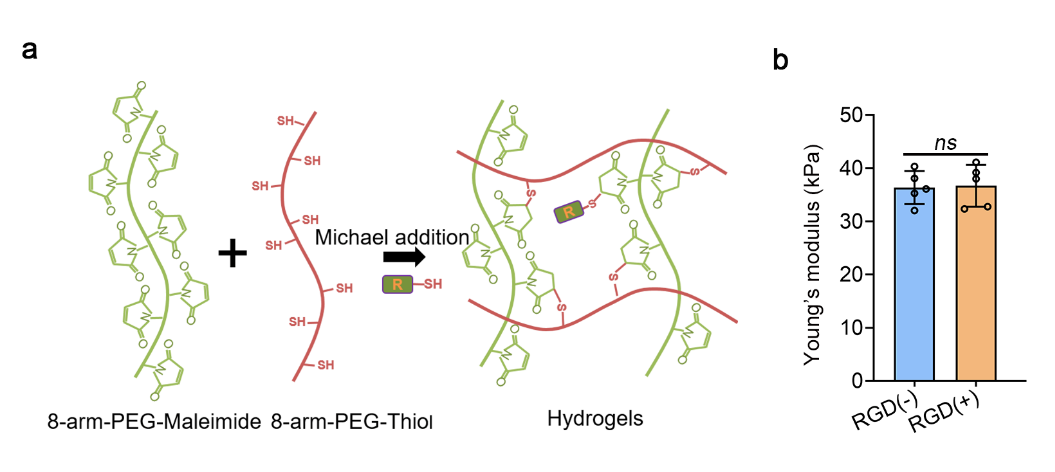


**Supplementary Fig. 9 Fabrication and Young’s moduli of PEG hydrogels.** **a**, The 8-arm PEG maleimide (PEG-MAL) and 8-arm PEG thiol (PEG-SH) were mixed to form PEG hydrogels by Michael addition reaction. Hydrogels were modified with RGD peptides to introduce adhesive domains. **b,** Young’s moduli of hydrogels synthesized without peptide or with peptide (RGD). (*n* = 5 samples per group). Results indicate that peptides modification did not alter the stiffness of PEG hydrogels. Data are presented as mean ± s.e.m., and *P* values were obtained using unpaired two-tailed Student’s t-test.

### Supplementary Figure 10


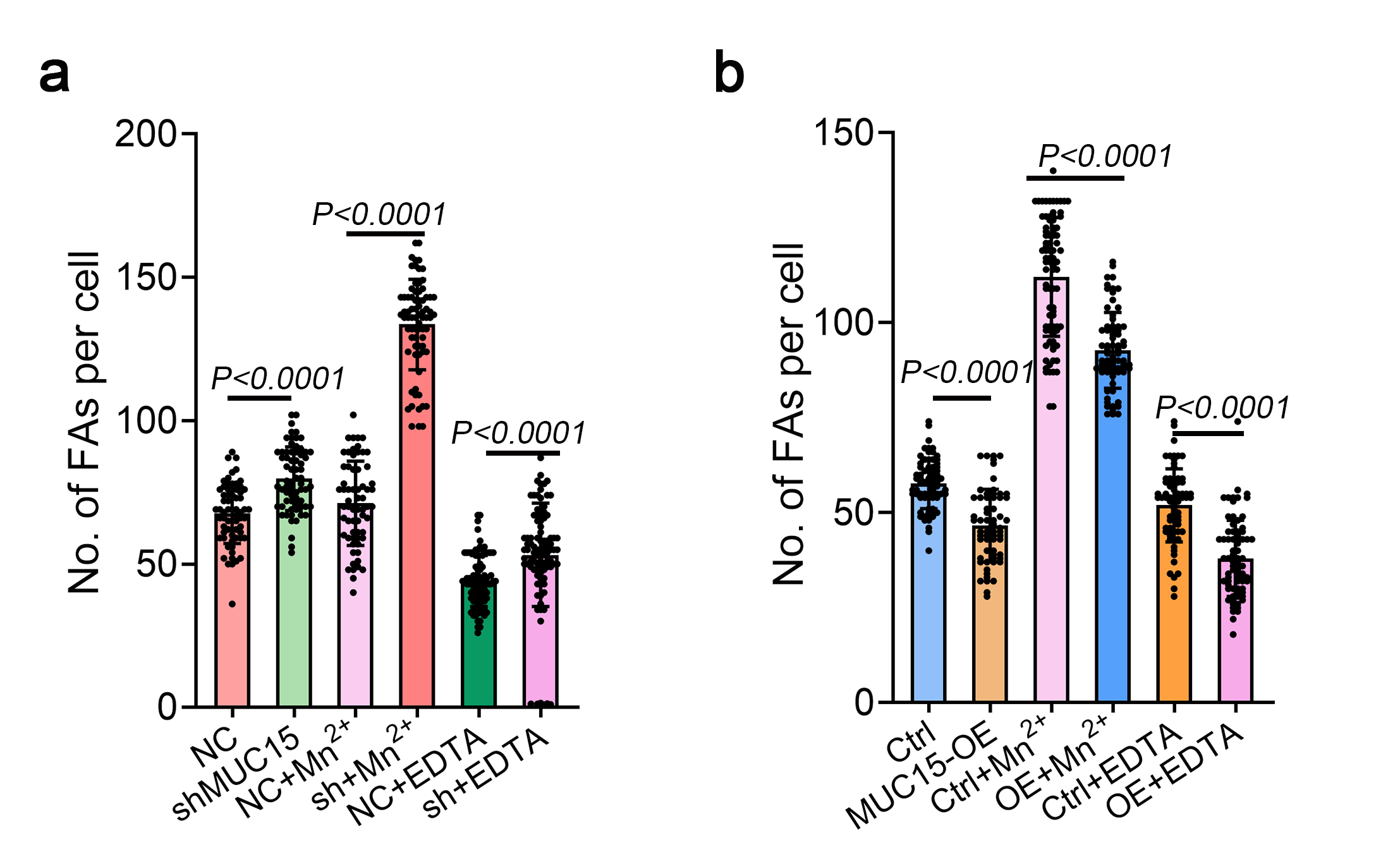


**Supplementary Fig. 10** **Focal adhesion counting analysis confirms MUC15's effects on integrin β1 activation. a**, **b**, Quantification of number of focal adhesions (FAs) per cell for conditions shown in **Figure 4a** and **4b**, respectively. Statistical analysis by one-way ANOVA.

### Supplementary Figure 11


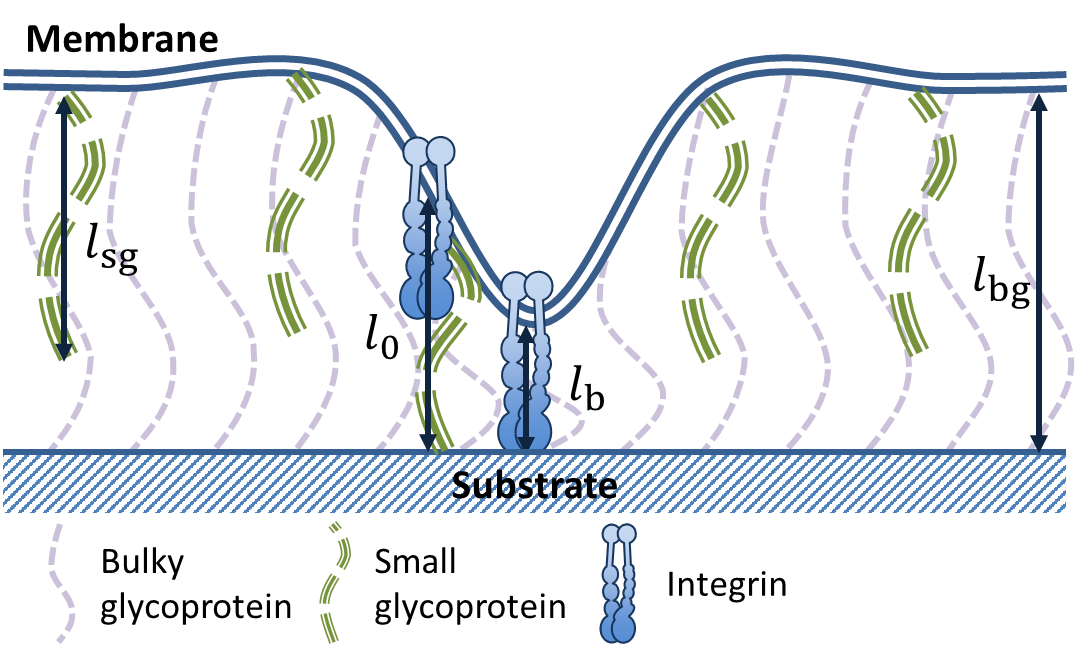


**Supplementary Fig. 11 An illustration labeling the various length scales used for calculating integrin binding.** $l_{\mathrm{bg}}$ is the equilibrium length of bulky glycoprotein, $l_{\mathrm{sg}}$ is the equilibrium length of small glycoprotein, $l_{b}$ is the equilibrium length of integrin-ligand bond, $l_{0}$ is the distance between the membrane and the substrate before integrin binding.

### Supplementary Figure 12


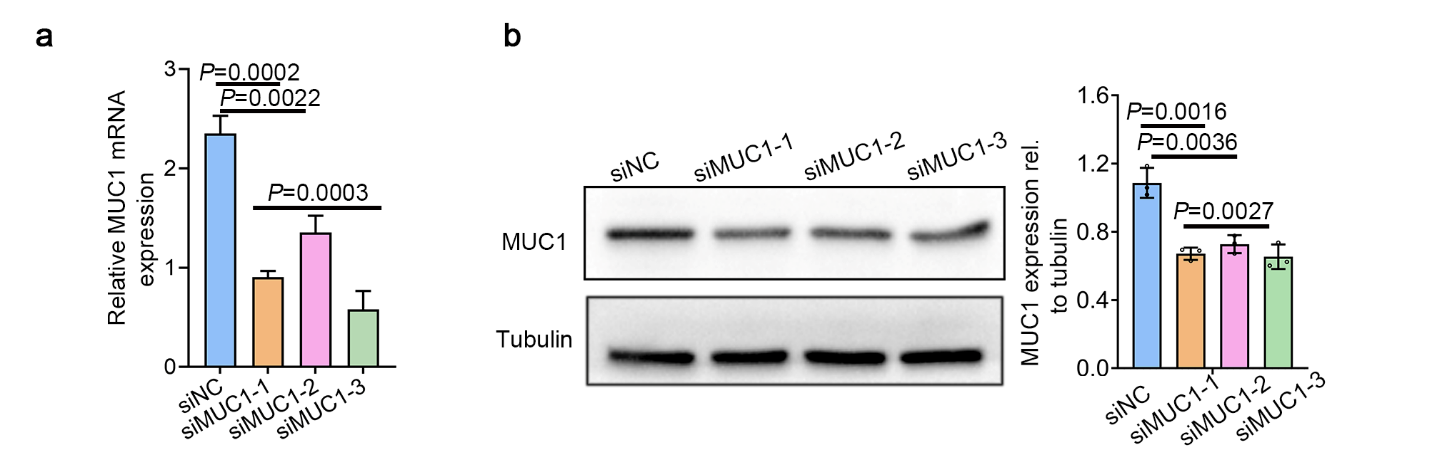


**Supplementary Fig. 12** **Characterization of MUC1 manipulation in MiaPaca-2 cells.** Results supporting the use of MiaPaca-2 cells as a model system for studying glycocalyx-dependent processes, as even their relatively low mucin levels can be effectively modulated. **a**, MUC1 mRNA levels in MiaPaca-2 cells measured by quantitative RT-PCR and normalized to 18S rRNA, demonstrating significant knockdown despite low baseline expression (*n* = 3 independent experiments). Statistical analysis by one-way ANOVA. **b**, Western blot analysis of MUC1 protein levels normalized to tubulin (left) with quantification relative to siNC control (right), showing that glycocalyx components can be effectively manipulated in MiaPaca-2 cells despite their lower baseline mucin expression. Data from three independent experiments; statistical analysis by one-way ANOVA.

### Supplementary Figure 13


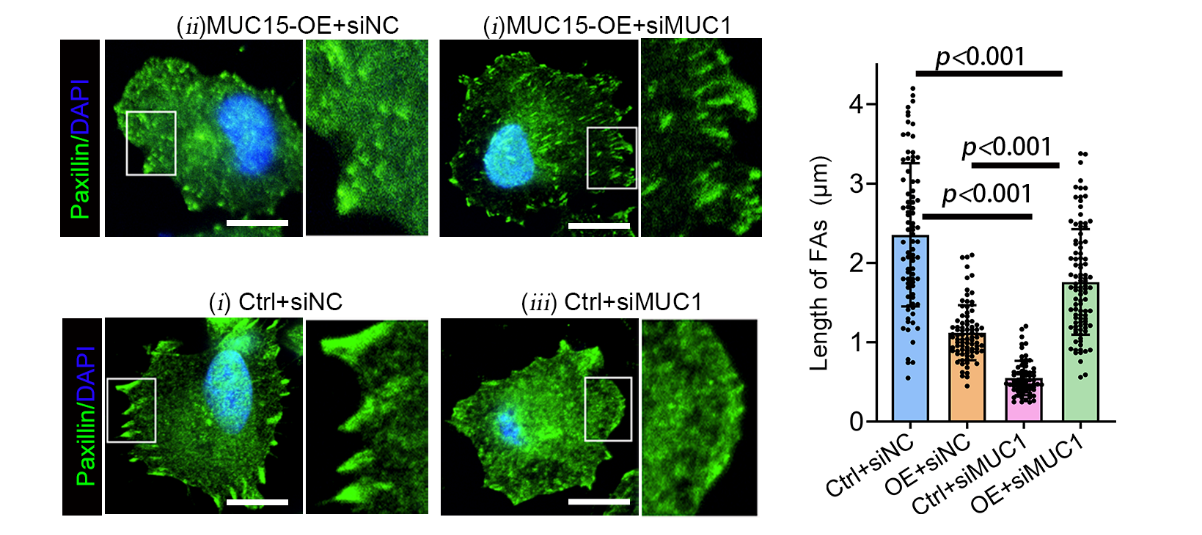


**Supplementary Fig. 13 Quantification of focal adhesion lengths in cells with different expression levels of glycoproteins (related to Fig. 5j).** **Left:** Fluorescence imaging of focal adhesion in cells with different expression levels of glycoproteins. **Right:** Corresponding quantification of focal adhesion lengths in cells with different expression levels of glycoproteins. Each data point represents individual cells from three independent experiments; *n* = 84 (Ctrl+siNC), 85 (OE+siNC), 68 (Crtl+siMUC1), and 100 (OE+siMUC1) cells, respectively. *P* values were obtained using one-way ANOVA. Scale bar: 50 μm.

### Supplementary Figure 14


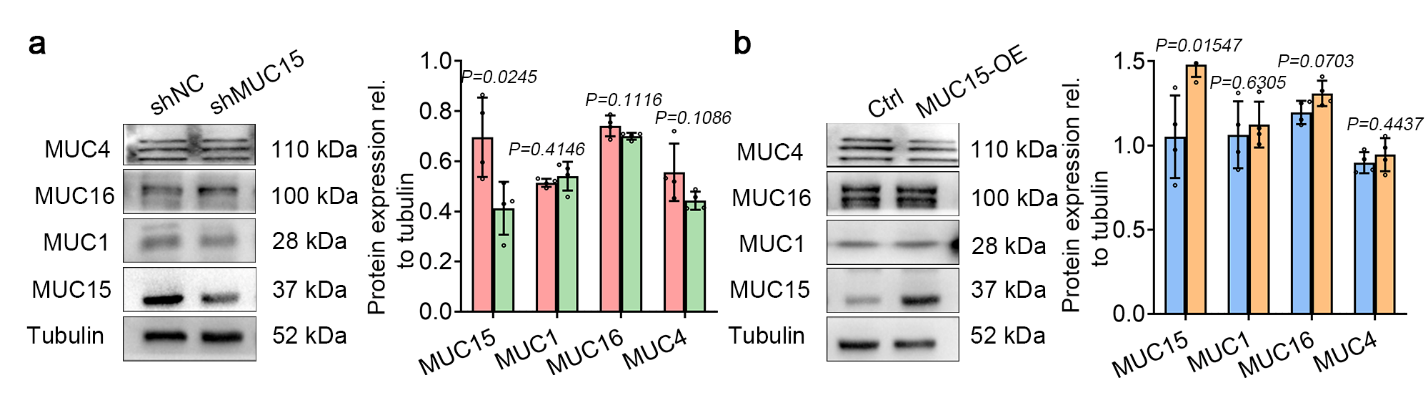


**Supplementary Fig. 14 The impact of MUC15 on composition and glycosylation of the glycocalyx**. **a**, Western blot analysis of MUC1, MUC4, and MUC16 in Panc-1 cells with and without MUC15 knockdown (left) with quantification normalized to tubulin and relative to shNC controls (right). **b**, Western blot analysis of MUC1, MUC4, and MUC16 in MiaPaca-2 cells with and without MUC15 overexpression (left) with quantification normalized to tubulin and relative to control cells (right). Data presented as mean ± s.e.m from three independent experiments. Statistical analysis by unpaired two-tailed Student's t-test revealed no significant differences in mucin expression levels (all *p* > 0.05), indicating that MUC15's effects occur independently of changes to other glycocalyx components.

### Supplementary Figure 15


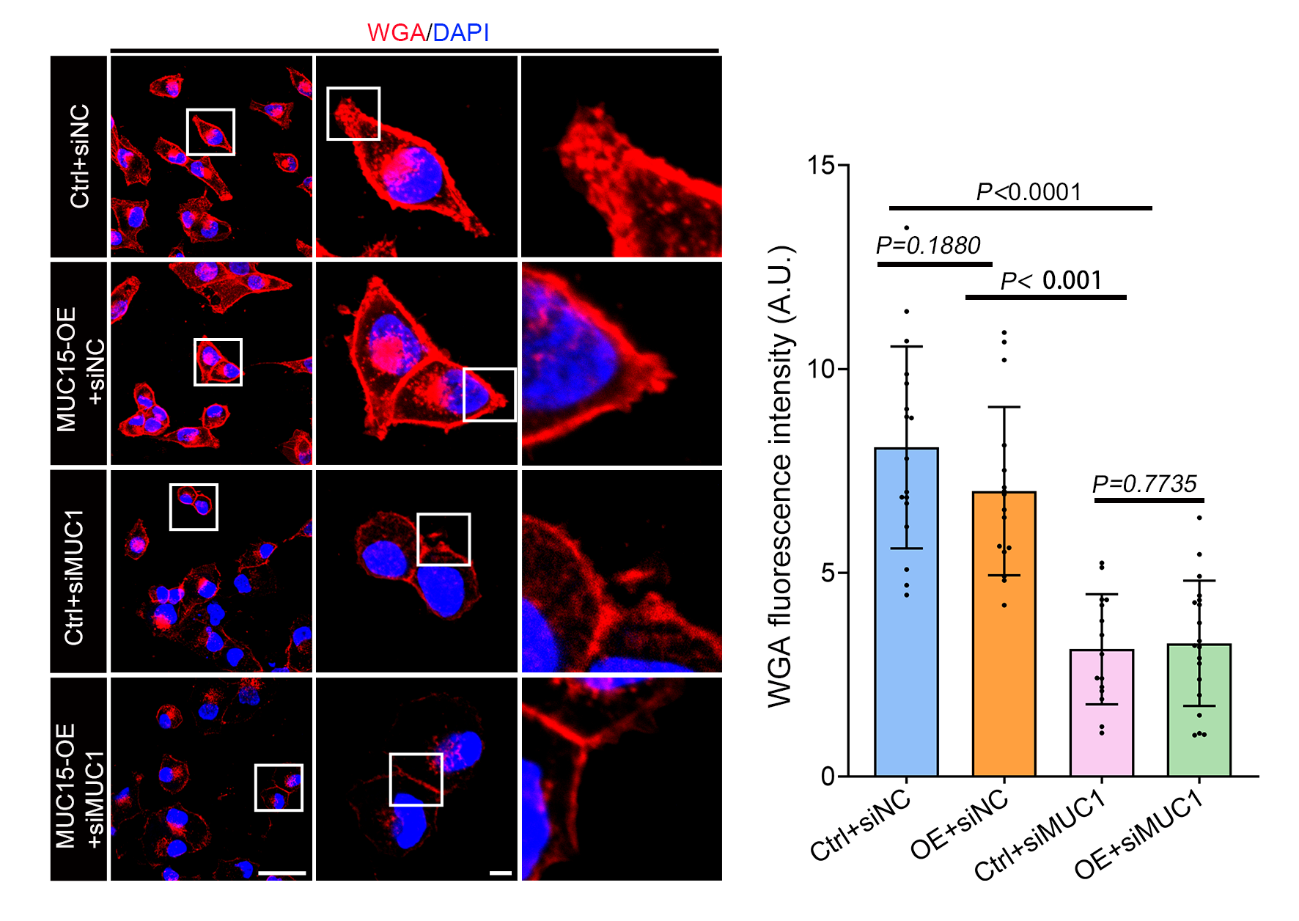


**Supplementary Fig. 15 Assessment of global glycocalyx structure using s-WGA lectin staining in MiaPaca-2 cells.** Immunofluorescence images showing s-WGA staining in control, MUC15-overexpressing, MUC1-knockdown, and MUC15-overexpressing plus MUC1-knockdown cells (left). MUC1 knockdown serves as a positive control for glycocalyx disruption. Quantification of s-WGA fluorescence intensity (right) reveals that while MUC1 knockdown significantly reduces glycosylation, MUC15 modulation does not affect overall glycocalyx structure. Data presented as mean ± s.e.m. (*n* = 50 cells per condition from three independent experiments). Statistical analysis by one-way ANOVA. Scale bar: 50 μm.

### Supplementary Figure 16


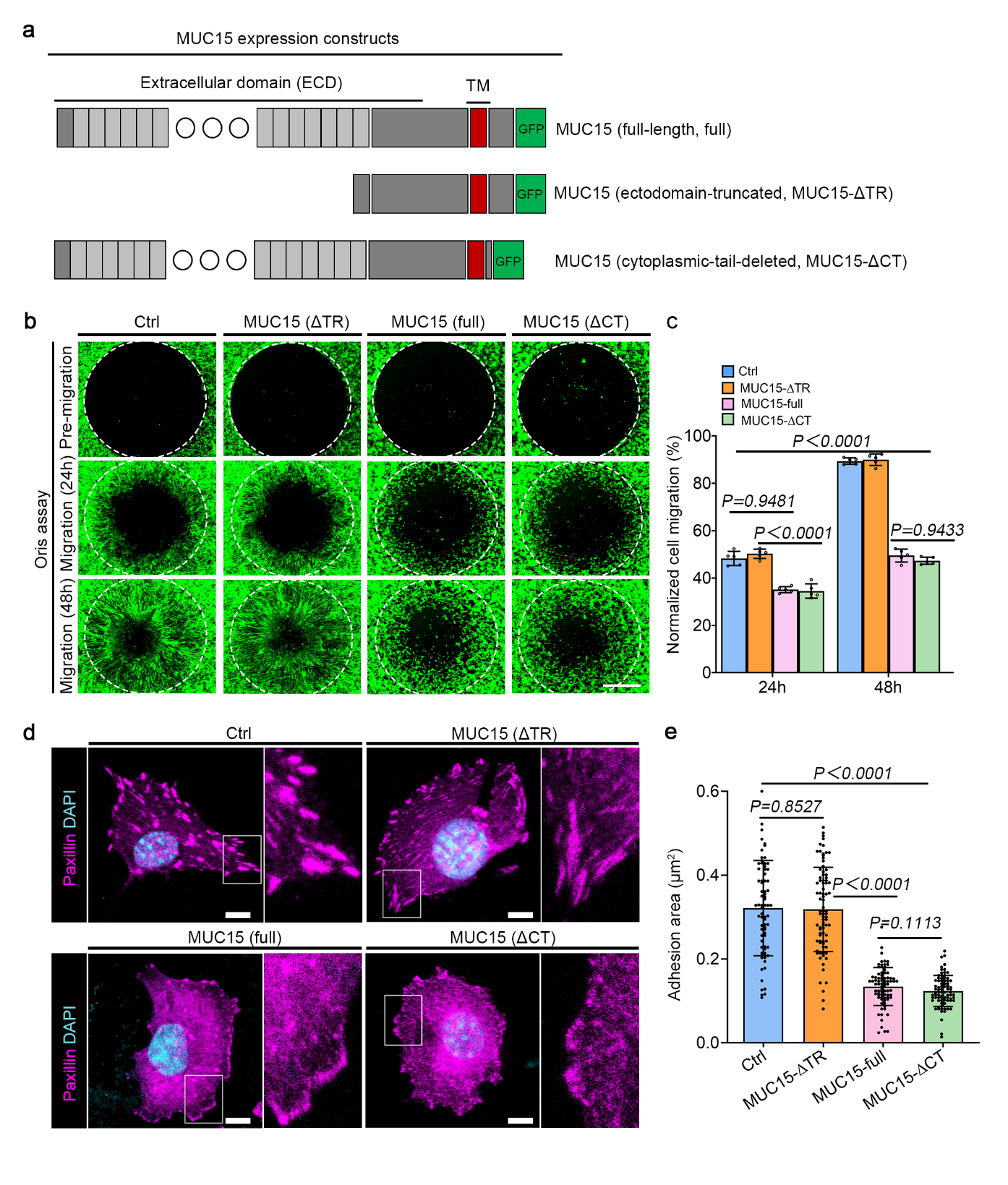
 Su**pplementary Fig. 16 Direct demonstration that MUC15's extracellular domain size controls cell migration and focal adhesion assembly. a.** Schematic representation of the three MUC15 variants engineered to isolate size-dependent effects: (1) full-length MUC15 (complete protein), (2) ectodomain-truncated MUC15 (MUC15-ΔTR, lacking the extracellular domain), and (3) cytoplasmic-tail-deleted MUC15 (MUC15-ΔCT, maintaining extracellular domain but lacking signaling capability). Each construct was fused with green fluorescent protein (GFP) to enable tracking of expression in transformed MiaPaCa-2 cells. **b.** Representative time-course images from Oris migration assays showing differential migration patterns of MiaPaCa-2 cells expressing each MUC15 variant (visualized by GFP). Dashed lines: initial migration boundary. **c.** Quantitative analysis of cell migration, measured as wound gap area relative to 0 h for conditions shown in panel **b**. Results demonstrate that only variants retaining the extracellular domain inhibit migration. Data presented as mean ± s.e.m. (*n* = 5 points from 3 independent samples per group; *p* values determined by one-way ANOVA with Tukey's post hoc test). **d.** Representative fluorescence images revealing focal adhesion patterns (marked by paxillin) in cells expressing each MUC15 variant, demonstrating size-dependent effects on adhesion assembly. **e.** Quantification of focal adhesion area across all variants, showing effects parallel to migration patterns. Data presented as mean ± s.e.m. (*n* = 75 (Ctrl), 85 (MUC15-ΔTR), 79 (MUC15-full) and 89 (MUC15-ΔCT) cells; *p* values determined by one-way ANOVA with Tukey's post hoc test). Scale bars: 100 μm (**b**), 50 μm (**d**).

### Supplementary Figure 17


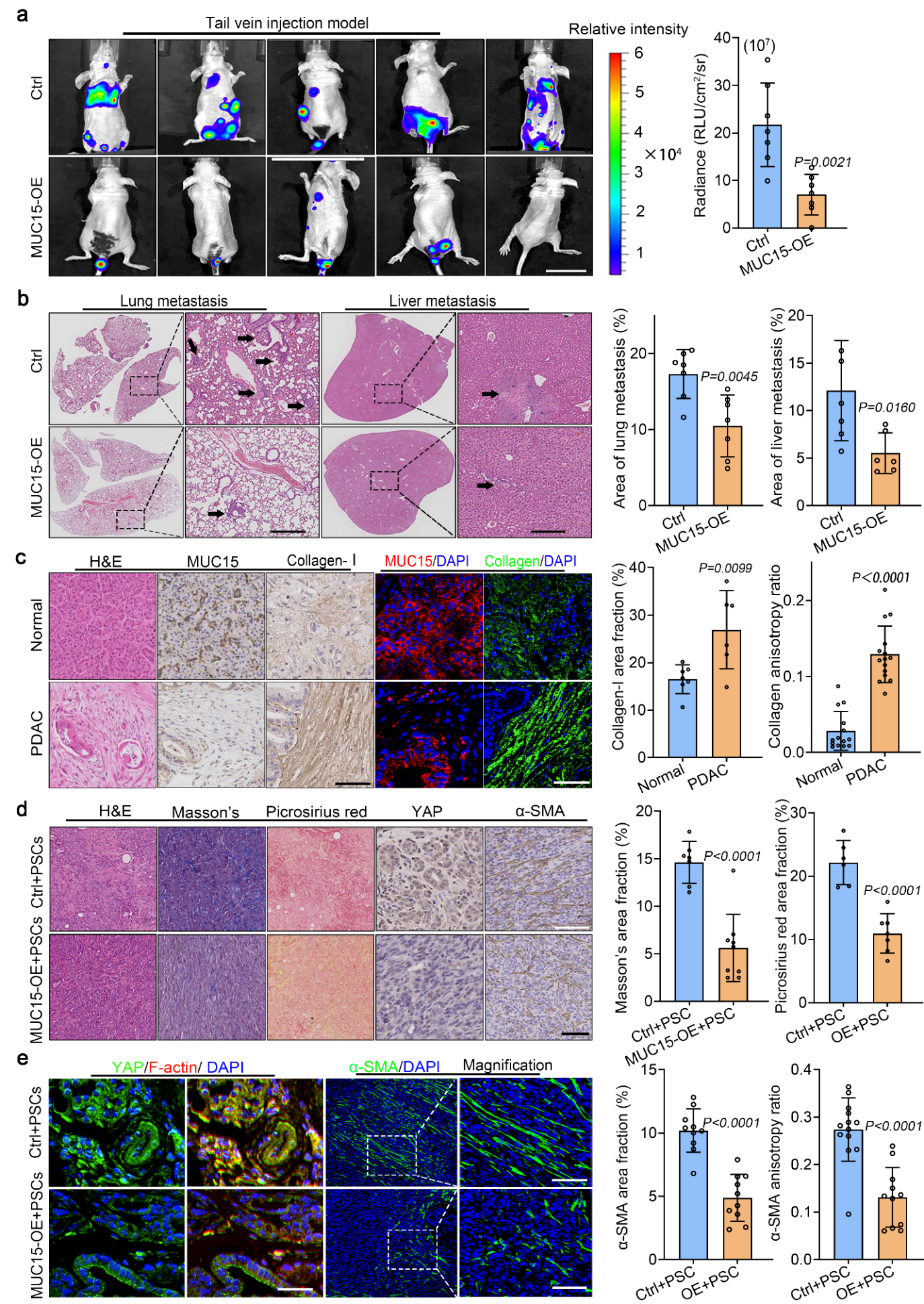


**Supplementary Fig. 17**  **Controlling the cell adhesion state by modulating MUC15 expression controls cancer metastasis and ECM remodeling in a mouse model of PDAC. a, Left:** Bioluminescence imaging shows that overexpression of MUC15 in MiaPaca-2 cells, which shifts cells from adhesion state (*i*) to adhesion state (*ii*) (c f. Fig. 5), inhibits metastasis when cells are injected via the tail-vein of nude mice *in vivo*. **Right**: Corresponding quantification of signal strength in mice injected with control or MUC15-overexpressing cells. **b,** H&E staining of lung and liver metastases (arrows) and quantification of spontaneous metastasis area. **c**, Representative H&E, immunohistochemistry and immunostaining of MUC15 and collagen Ι in normal and PDAC tissues. Quantification of collagen coverage area and anisotropy ratio. **d,** Co-implantation models: subcutaneous injection of cell mixtures containing MUC15-control or MUC15-overexpressing MiaPaca-2 cells into BALB/c nude mice. Tumor sections subjected to H&E staining, picrosirius red and Masson’s staining for fibrillar collagens, and YAP and α-SMA staining. Quantification of fibrillar collagen coverage area and anisotropy ratio. **e**, Immunostaining of YAP and α-SMA in tumor sections. Zoomed regions display details of α-SMA alignment. Quantification of the α-SMA coverage area and anisotropy ratio. Each data point represents an individual mouse. *n* = 10 mice. Each data point represents an independent experiment. Statistical analyses were performed using unpaired two-tailed Student’s t-test. Scale bars: 10 mm (**a**) and 100 μm (**b**, **c**, **d**, **e**).

### Supplementary Figure 18


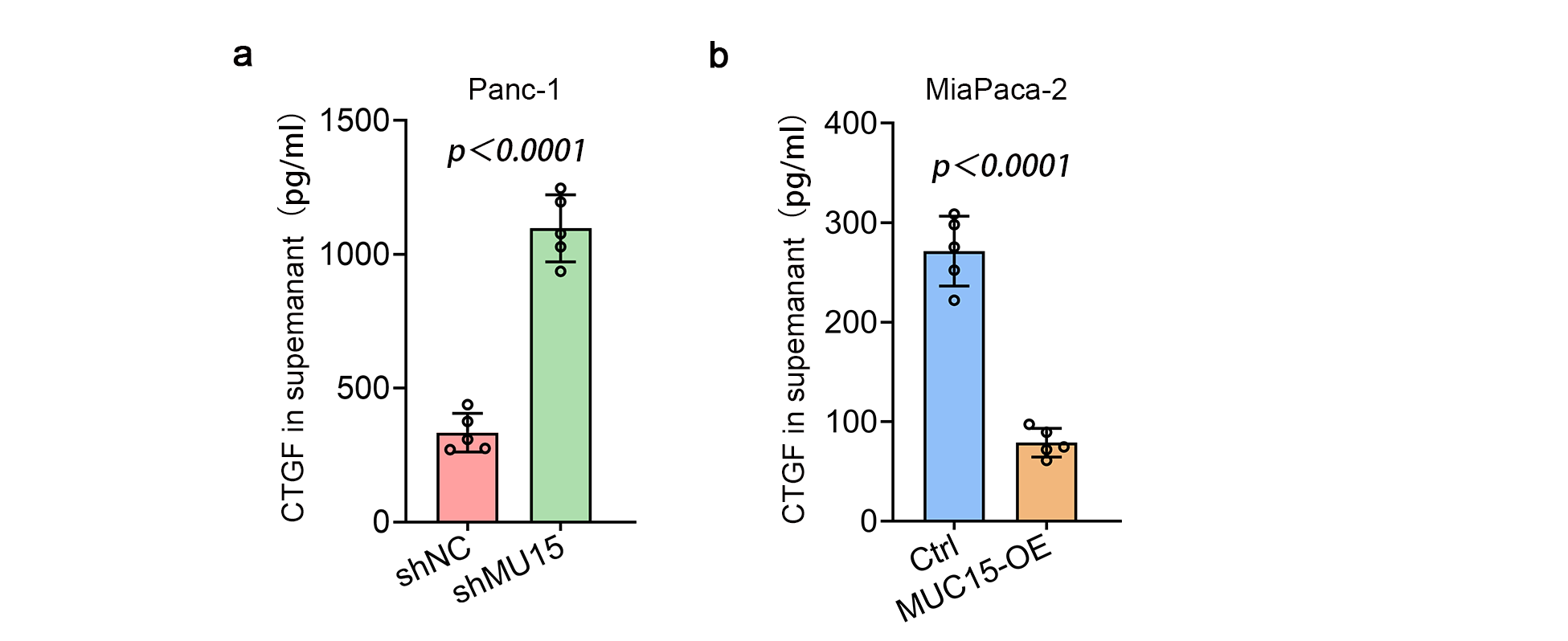


**Supplementary Fig. 18 MUC15 regulates CTGF secretion**. ELISA was used to quantify the CTGF secretion levels in Panc-1 and MiaPaca-2 cells with MUC15 depletion and overexpression (*n* = 5 experiments per group). *P* values were obtained using unpaired two-tailed Student’s t-test.

### Supplementary Figure 19


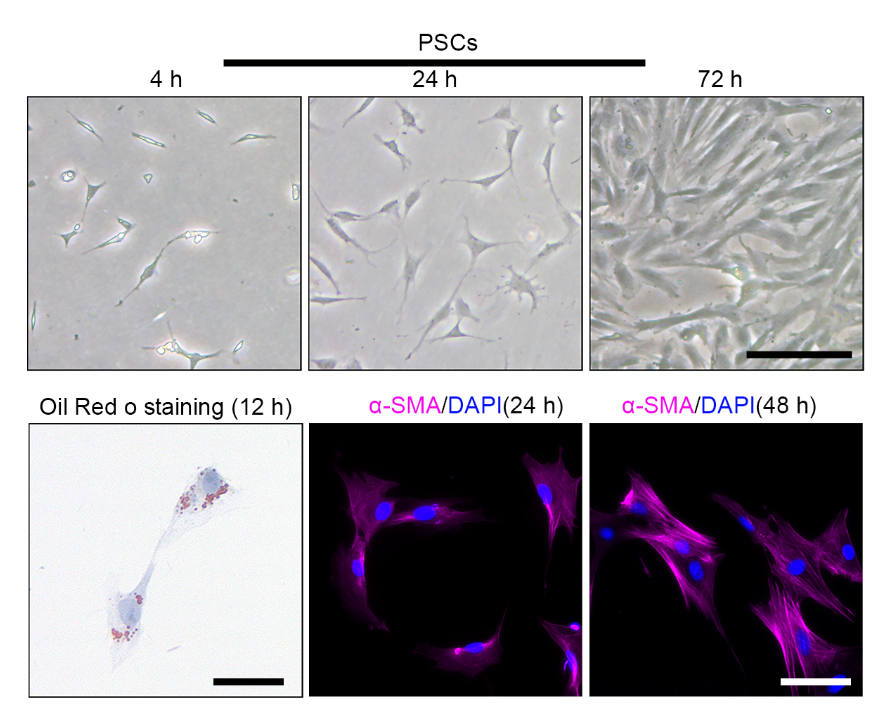


**Supplementary Fig. 19** **Assessment of pancreatic stellate cell (PSC) activation.** **Top**: Representative images of primary stellate cells after isolated for 4, 24 and 72 h cultured in the plate. **Bottom**: Oil Red O staining of intracellular fat droplets (12 h) and immunostaining of α-smooth muscle actin (24 h and 72 h). Scale bars: 50 μm (top) and 20 μm (bottom).

### Supplementary Figure 20


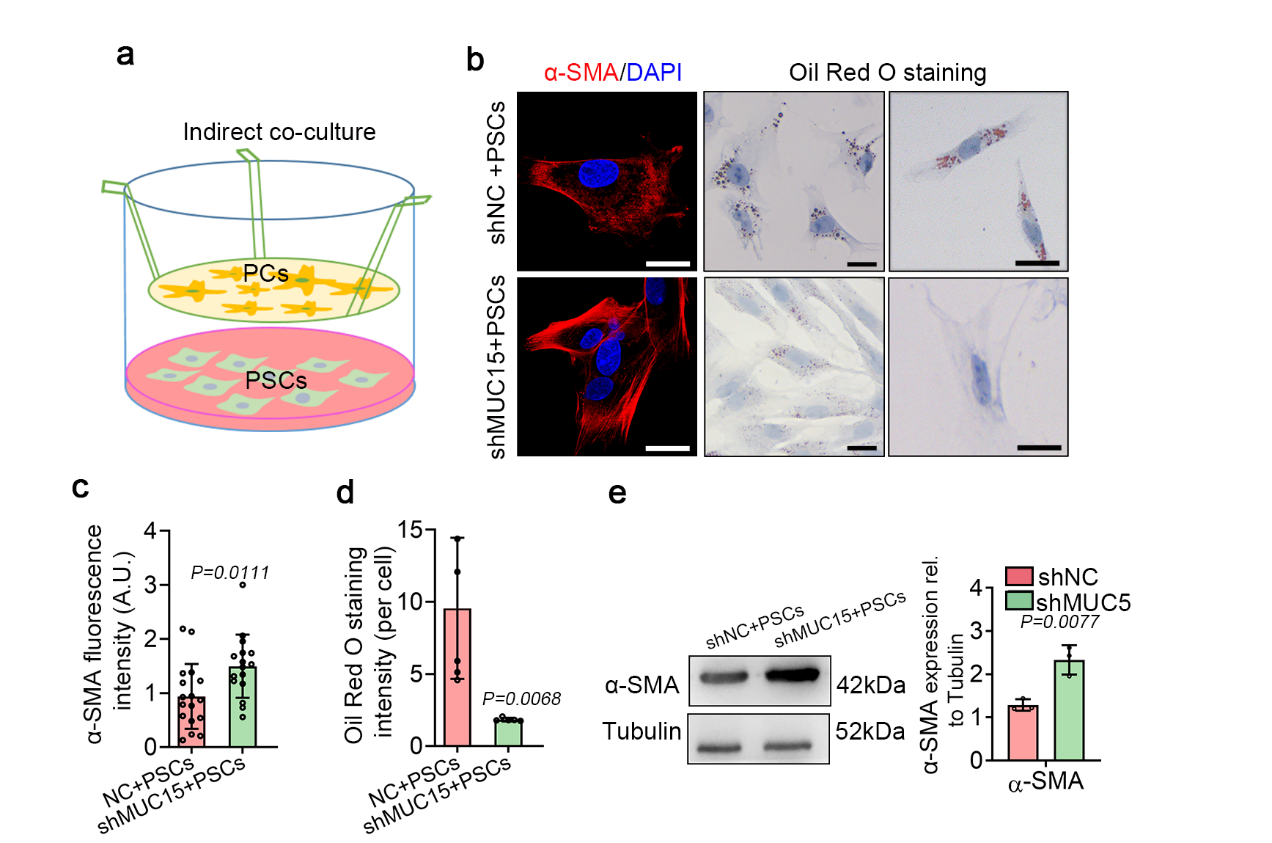


**Supplementary Fig. 20** **MUC15 dependent paracrine effect on PSC activation**. **a**, Schematic diagram of co-culture of pancreatic cancer cells and PSCs. **b**, Representative images of immunostaining of PSCs for a-SMA and oil red o staining when cultured with cancer cells with or without MUC15 knockdown. Scale bars: 50 μm (left) and 20 μm (right) in zoom region. **c, d,** Quantification of α-SMA fluorescence intensity and oil red o staining intensity for conditions indicated in panel **b, e,** Immunoblotting analysis of α-SMA when cultured with cancer cells with or without MUC15 knock down and the corresponding quantification of α-SMA relative to control cells. Each data point represents an independent experiment; *n* = 3 independent experiments. Data are presented as mean ± s.e.m., and *P* values were obtained using unpaired two-tailed Student’s t-test.

Supplementary Tables

Table S1. Mapping of Adhesion States to Model and Experiments

| **Adhesion State** | **Model Parameters** | **Experimental Manipulation** | **Integrin clustering and focal adhesion assembly** |
| --- | --- | --- | --- |
| **State (i)** | Low $k_{on0}$; $p_{\mathrm{sg}}=0\%$ | Control MiaPaca-2 cells | Stable integrin clustering; large focal adhesions |
| **State (ii)** | Low $k_{on0}$; $p_{\mathrm{sg}}=100\%$ | MUC15 overexpression in MiaPaca-2 cells | Inhibited integrin clustering; small/immature focal adhesions |
| **State (iii)** | High $k_{on0}$; $p_{\mathrm{sg}}=0\%$ | MUC1 knockdown in MiaPaca-2 cells | Random integrin binding; loss of clustering; weak focal adhesions |
| **State (i)** | High $k_{on0}$; $p_{\mathrm{sg}}=100\%$ | MUC15 overexpression in MUC1-depleted MiaPaca-2 cells | Recovered integrin clustering; restored focal adhesion assembly |

Table S2. Reagent or resource

| **Antibodies** | **Source** | **Identifier** |
| --- | --- | --- |
| Rabbit anti-MUC15 antibody | Sigma-Aldrich | Cat#HPA026110 |
| Mouse anti-Integrin β1 antibody | abcam | Cat#ab30394 |
| Mouse anti-Paxillin antibody | BD Biosciences | Cat#610051 |
| Mouse anti-Phospho-FAK antibody | MilliporeSigma | Cat#05-1140 |
| Rabbit anti-Phospho-Paxillin antibody | abcam | Cat#ab4833 |
| Rabbit anti-Integrin β1 antibody | abcam | Cat#ab183666 |
| Mouse act-Integrinβ1 antibody | MilliporeSigma | Cat#MAB2079Z |
| Rabbit anti-Phospho-FAK antibody | Cell Signaling Technology | Cat#8566 |
| Rabbit anti-Paxillin antibody | abcam | Cat#ab32084 |
| Rabbit anti-FAK antibody | Cell Signaling Technology | Cat#71433 |
| Rabbit anti-Phospho-Myosin Light chain 2 antibody | Cell Signaling Technology | Cat#3671 |
| Rabbit anti-Myosin Light chain 2 antibody | Cell Signaling Technology | Cat#3672 |
| Rabbit anti-Phospho-Cofilin（Ser3）antibody | Cell Signaling Technology | Cat#3311 |
| Rabbit anti-Cofilin antibody | Cell Signaling Technology | Cat#5175 |
| Anti-mouse IgG (H+L), (Alexa Fluor 647 Conjugate) | Cell Signaling Technology | Cat#4410 |
| Anti-Rabbit IgG (H+L), (Alexa Fluor 647 Conjugate) | Cell Signaling Technology | Cat#4414 |
| Anti-mouse IgG (H+L), (Alexa Fluor 488 Conjugate) | Cell Signaling Technology | Cat#4408 |
| Anti-Rabbit IgG (H+L), (Alexa Fluor 488 Conjugate) | Cell Signaling Technology | Cat#4412 |
| Anti-Rabbit IgG (H+L), (Alexa Fluor 594 Conjugate) | Cell Signaling Technology | Cat#8889 |
| Anti-Mouse IgG (H+L), (Alexa Fluor 594 Conjugate) | Cell Signaling Technology | Cat#8890 |
| Alexa Fluor 555 Phalloidin | Cell Signaling Technology | Cat#8953 |
| Rabbit anti-YAP antibody | Cell Signaling Technology | Cat#14074 |
| Rabbit anti-Phospho-YAP（Ser127） antibody | Cell Signaling Technology | Cat#13008) |
| Mouse anti-LATS1/2 antibody | Immunoway | Cat# YP6125 |
| Mouse Phospho- LATS1/2 antibody | Immunoway | Cat# YP1047 |
| Rabbit anti-Phospho-YAP（Ser127） antibody | Cell Signaling Technology | Cat#13008) |
| Mouse anti-LATS1/2 antibody | Immunoway | Cat# YP6125 |
| Mouse Phospho- LATS1/2 antibody | Immunoway | Cat# YP1047 |
| Rabbit anti-GAPDH antibody | Cell Signaling Technology | Cat#2118) |
| Mouse anti-α-Tublin antibody | Proteintech | Cat# 66031-1-1g |
| Rabbit Anti-IgG, HRP-Linked | Cell Signaling Technology | Cat# 7074) |

Table S3. List of input parameter values.

| **Symbol** | **Parameter Description** | **Value** | **Ref** |
| --- | --- | --- | --- |
| $k_{\mathrm{act}}$ | Activation rates of integrin | 0.5/s | ^3^ |
| $k_{\mathrm{deact}}$ | Deactivation rates of integrin | 5/s | ^3^ |
| $l_{\mathrm{bg}}$ | Equilibrium length of glycocalyx layer without force | 50 nm | ^3, 6^ |
| $l_{b}$ | Equilibrium length of integrin-ligand bond without force | 27 nm | ^3^ |
| $l_{\mathrm{sg}}$ | Equilibrium length of small mucins without force | 40 nm | - |
| $\sigma_{m}$ | Spring constant of cell membrane | 0.4 pN/nm | ^3^ |
| $\sigma_{\mathrm{sub}}$ | Spring constant of substrate | 400 pN/nm | ^3^ |
| $\sigma_{b}$ | Spring constant of integrin-ligand bond | 2 pN/nm | ^3^ |
| $\sigma_{\mathrm{bg}}$ | Spring constant of glycocalyx layer | 0.2 pN/nm | ^3^ |
| $\sigma_{\mathrm{sg}}$ | Spring constant of small mucins | 0.2 pN/nm | - |
| $D_{\mathrm{int}}$ | Diffusion coefficient of integrin | 10^4^ nm^2^/s | ^3, 7^ |
| $D_{\mathrm{sg}}$ | Diffusion coefficient of small glycoprotein | 10^2^ nm^2^/s | - |
| $k_{\mathrm{on}0}$ | intrinsic binding rate | 10^12^~10^16^/s | - |
| $k_{off0}$ | intrinsic unbinding rate | 10^-3^/s | - |
| $k_{B}T$ | Thermal energy | 4.28 pN·nm | ^3^ |
| $n_{\mathrm{int}}$ | Total integrin number on the calculated area | 1000 | - |
| $F_{0}$ | Characteristic rupture force of integrin-ligand bond | 2 pN | ^5^ |

Supplementary Reference

1. Paszek MJ*, et al.* The cancer glycocalyx mechanically primes integrin-mediated growth and survival. *Nature* **511**, 319-325 (2014).

2. Lee D-H, Choi S, Park Y, Jin H-s. Mucin1 and Mucin16: Therapeutic Targets for Cancer Therapy. *Pharmaceuticals* **14**, 1053 (2021).

3. Paszek MJ, Boettiger D, Weaver VM, Hammer DA. Integrin clustering is driven by mechanical resistance from the glycocalyx and the substrate. *PLoS Comput Biol* **5**, e1000604 (2009).

4. Xu G-K, Qian J, Hu J. The glycocalyx promotes cooperative binding and clustering of adhesion receptors. *Soft Matter* **12**, 4572-4583 (2016).

5. Chan CE, Odde DJ. Traction Dynamics of Filopodia on Compliant Substrates. *Science* **322**, 1687-1691 (2008).

6. Möckl L*, et al.* Quantitative Super-Resolution Microscopy of the Mammalian Glycocalyx. *Dev Cell* **50**, 57-72 (2019).

7. Yuan J-W*, et al.* Diffusion Behaviors of Integrins in Single Cells Altered by Epithelial to Mesenchymal Transition. *Small* **18**, 2270023 (2022).
